# Hydro‐Sensitive, In Situ Ultrafast Physical Self‐Gelatinizing, and Red Blood Cells Strengthened Hemostatic Adhesive Powder with Antibiosis and Immunoregulation for Wound Repair

**DOI:** 10.1002/advs.202306528

**Published:** 2023-11-30

**Authors:** Lingling Shang, Yonggan Yan, Zhao Li, Hong Liu, Shaohua Ge, Baojin Ma

**Affiliations:** ^1^ Department of Periodontology & Tissue Engineering and Regeneration School and Hospital of Stomatology Cheeloo College of Medicine Shandong University Jinan Shandong 250012 China; ^2^ Shandong Key Laboratory of Oral Tissue Regeneration & Shandong Engineering Laboratory for Dental Materials and Oral Tissue Regeneration Jinan Shandong 250012 China; ^3^ Shandong Provincial Clinical Research Center for Oral Diseases Jinan Shandong 250012 China; ^4^ State Key Laboratory of Crystal Materials Shandong University Jinan Shandong 250013 China

**Keywords:** adhesive, collagen, hemostatic powder, tannic acid, wound repair

## Abstract

Immediate and effective hemostatic treatments for complex bleeding wounds are an urgent clinical demand. Hemostatic materials with characteristics of adhesion, sealing, anti‐infection, and concrescence promotion have drawn growing concerns. However, pure natural multifunctional hemostatic materials with in situ ultrafast self‐gelation are rarely reported. In this study, a hydro‐sensitive collagen/tannic acid (ColTA) natural hemostatic powder is developed that can in situ self‐gel to form adhesive by the non‐covalent crosslinking between tannic acid (TA) and collagen (Col) in liquids. The physical interactions endow ColTA adhesive with the characteristics of instantaneous formation and high adhesion at various substrate surfaces. Crucially, ColTA powder adhesive shows an enhanced adhesion performance in the presence of blood due to the electrostatic interactions between ColTA adhesive and red blood cells, conducive to effective in situ sealing and rapid hemostasis. The biocompatible and hemocompatible ColTA adhesive can effectively control bleeding and seal the wounds of the caudal vein, liver, heart, and femoral arteries in rats. Furthermore, the low‐cost and ready‐to‐use ColTA adhesive powder also possesses good antibacterial and inhibiting biofilm formation ability, and can efficiently regulate immune response by the NF‐κB pathway to promote wound repair, making it a highly promising hemostatic material with great potential for biomedical applications.

## Graphical abstract

A hydro‐sensitive collagen/tannic acid (ColTA) natural hemostatic powder that can in situ self‐gel to form adhesive by the non‐covalent crosslinking. ColTA powder adhesive shows a red blood cell‐enhanced adhesion performance to effectively control bleeding and seal the wounds and possesses good antibacterial and inhibiting biofilm formation ability, and immune regulation by the NF‐κB pathway to promote wound repair.

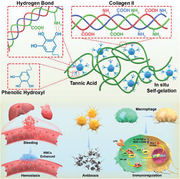

## 1 Introduction

Acute, excessive bleeding, associated with high mortality, is a serious complication during major trauma or surgery scenarios, sometimes bringing about organ failure and hemorrhagic shock. Immediate and effective hemostatic treatments are necessary to prevent life‐threatening emergencies, due to the unbalance between the body's coagulation function and the heavy bleeding.^[^ ^1^, ^2^, ^3^ ^]^ Common methods of hemostasis for superficial or extremity bleeding are already quite mature, such as finger compression, pressure dressing, and tourniquets. However, the hemostasis of non‐compressible internal or irregularly shaped bleeding remains inadequate, especially for rapid hemostasis before reaching the hospital.^[^ ^4^ ^]^ In addition, injury bleeding with infection and tissue damage such as burn surgery bleeding have anti‐infectious and regenerative requirements.^[^ ^5^ ^]^ Therefore, hemostatic materials with multifunctional characteristics, including rapid hemostasis, good adhesion, favorable mechanical performances to serve as the maskant of the bleeding site keeping blood pressure, eligible biocompatibility, fine anti‐infection, and concrescence promotion capacities, should be developed to follow the increasingly urgent clinical demand.^[^ ^6^ ^]^

In addition to the traditional gauzes, sponges, and tourniquets, various forms of hemostatic materials, made from natural polymers (polysaccharide), synthetic materials (polylactic acid), or inorganic species (kaolin), come into existence with the progress of material preparation technology, including hydrogels, adhesives, hemostatic powders, and styptics.^[^ ^7^, ^8^ ^]^ Different hemostatic materials with their specific merits are suitable for handling diverse types of bleeding. The common choices are gauzes, sponges, and tourniquets, among which the former two stop bleeding mainly by absorbing liquid to augment blood viscosity and concentration of clotting factors, swelling to cover the wound, or rapidly aggregating platelets and erythrocytes to further accelerate hemostasis (cellulose‐based soluble gauze).^[^ ^6^, ^7^ ^]^ Tourniquets are used to pressure blood vessels to block blood flow for hemostasis, which applies to excessive bleeding from extremities. However, the frequent replacement of gauze can cause suffering due to secondary damage from sticking to the wound surface. The misuse of tourniquets can bring about peripheral nerve injury, local skin damage, and even limb necrosis. Crucially, these options consistently require to be compressed or fixed at the bleeding site, which is unsatisfactory for incompressible internal or irregularly shaped hemorrhage such as bleeding in the heart, liver, and brain.^[^ ^7^ ^]^ Various natural or synthetic material‐based hemostatic hydrogels are developed for incompressible, irregular, deep, narrow, or perforating wounds owing to their excellent liquidity, plasticity, and shape memory properties.^[^ ^9^, ^10^ ^]^ Despite the rapid development of adhesive hydrogels, the issues of wet adhesion properties to achieve rapid tissue adhesion and hemostasis under the flowing blood still need to be well addressed.^[^ ^11^, ^12^ ^]^ Hemostatic powders composed of tiny particles can fully cover and permeate into complex, narrow, deep, and incompressible bleeding wounds to acquire rapid hemostasis within a short time. However, the lightweight hemostatic powders cannot stick firmly to severe bleeding wounds to achieve the sealing effect, hindering the hemostatic effect. Some hemostatic powders can dissolve or spread into the blood, which might aggrandize the risk of vascular embolization.^[^ ^6^, ^7^ ^]^

To address the above‐mentioned issues, recently, self‐gelling powders have been developed, with the characteristic of rapidly forming in situ hydrogels upon hydration and sealing the bleeding wound.^[^ ^6^, ^13^, ^14^ ^]^ Recent studies reported that self‐gelling powders based on polyacrylic acid/polyacrylamide/quaternate chitosan or polyethyleneimine/polyacrylic acid/quaternized chitosan, can form stable and adhesive hydrogels by physical crosslinking between polymers and stop bleeding in a short time.^[^ ^6^, ^14^ ^]^ Despite the characteristics of quick water absorption and gelatinization, the main compositions of powders are synthetic polymers with the peculiarities of long degradation time, acid degradation products, and lack of biological activities. Natural polymers are highly biocompatible and biodegradable, with a relatively short degradation period without any acidic degradation products, and various superior biological activities.^[^ ^7^ ^]^ Natural polymers type II collagen (Col‐II) is the cartilaginous extracellular matrix (ECM) molecule, existing as a fibril with the basic triple helical structure in the cartilage. Col‐II has been widely applied to diverse fields, including food processing, osteochondral tissue engineering, the treatment of osteoarthritis, and even pharmaceutical and industrial (shampoo and lipstick) applications.^[^ ^15^, ^16^ ^]^ A recent study also demonstrated that Col‐II from cartilage can alleviate inflammation and facilitate ECM deposition in wound healing.^[^ ^17^ ^]^ However, its potential role in hemostasis has been less reported. A potentially feasible strategy is to incorporate Col‐II polymers with polyphenols to achieve tissue adhesion for hemostasis. Tannic acid (TA), a natural polyphenol with the properties of anti‐inflammation, antimicrobial activity, and tissue adhesion, has been studied as a natural crosslinker to interact with biomacromolecules such as proteins and polysaccharides via the strong noncovalent bonds, mainly hydrogen bonding, and induce the gelation of biomacromolecules.^[^ ^18^, ^19^ ^]^ The phenolic hydroxyl groups of TA provide hydrogen donors or acceptors for hydrogen bond formation in the polyphenol‐protein reaction mechanism.^[^ ^19^, ^20^ ^]^ Thus, the Col‐II polymers incorporating TA could be a reasonable choice for fabricating powder adhesives.

**Figure 1:**
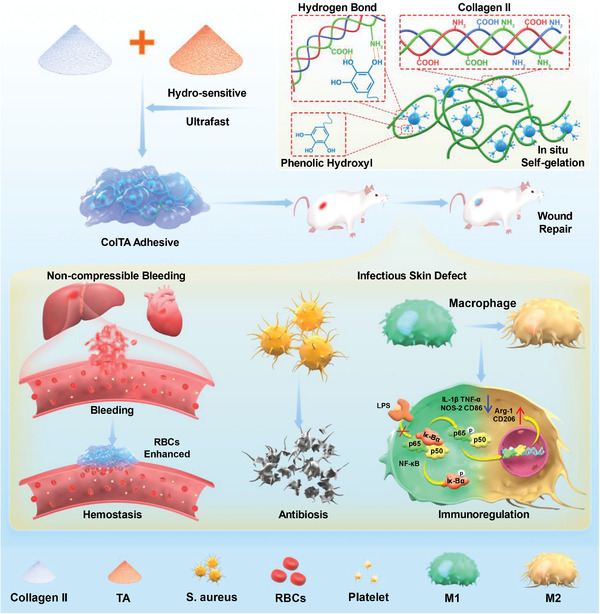
Schematics for the hydro‐sensitive, in situ ultrafast physical self‐gelatinizing, red blood cells strengthened ColTA hemostasis adhesive with the characteristics of antibiosis and immunoregulation for wound repair.

In this work, we report a hydro‐sensitive and ultrafast in situ self‐gelled collagen/tannic acid (ColTA) natural hemostatic adhesive powder by the crosslinking between Col‐II and TA in wet conditions. Upon mixing with aqueous solutions, ColTA hemostatic powder can rapidly form adhesive due to the non‐covalent interactions between Col‐II and TA, resulting in excellent adhesion performance on various materials. Especially for blood, ColTA powder can absorb blood quickly and self‐gel, thereby concentrating the coagulation factors, which can effectively exercise the function of hemostasis and seal the bleeding sites. Furthermore, our results demonstrate that the biocompatible and hemocompatible ColTA powder is effective in controlling bleeding of irregularly shaped and incompressible wounds, such as bleeding of the caudal vein, liver, heart, and femoral arteries in rats. Importantly, compared with other aqueous conditions (water or normal saline), the self‐assembling ColTA powder possesses red blood cells (RBCs)‐strengthened adhesion properties, which is conducive to rapid hemostasis. In addition, the accessible, low‐cost, and user‐friendly ColTA powder also has the abilities of antibiosis, immunomodulation, and wound repair promotion, endowing the ColTA powder with great potentiality for hemostatic, anti‐infectious, and conglutinant applications in the biomedical field (**Figure** **1**).

## 2 Results and Discussion

### 2.1 Preparation and Characterization of the ColTA Adhesive

**Figure 2:**
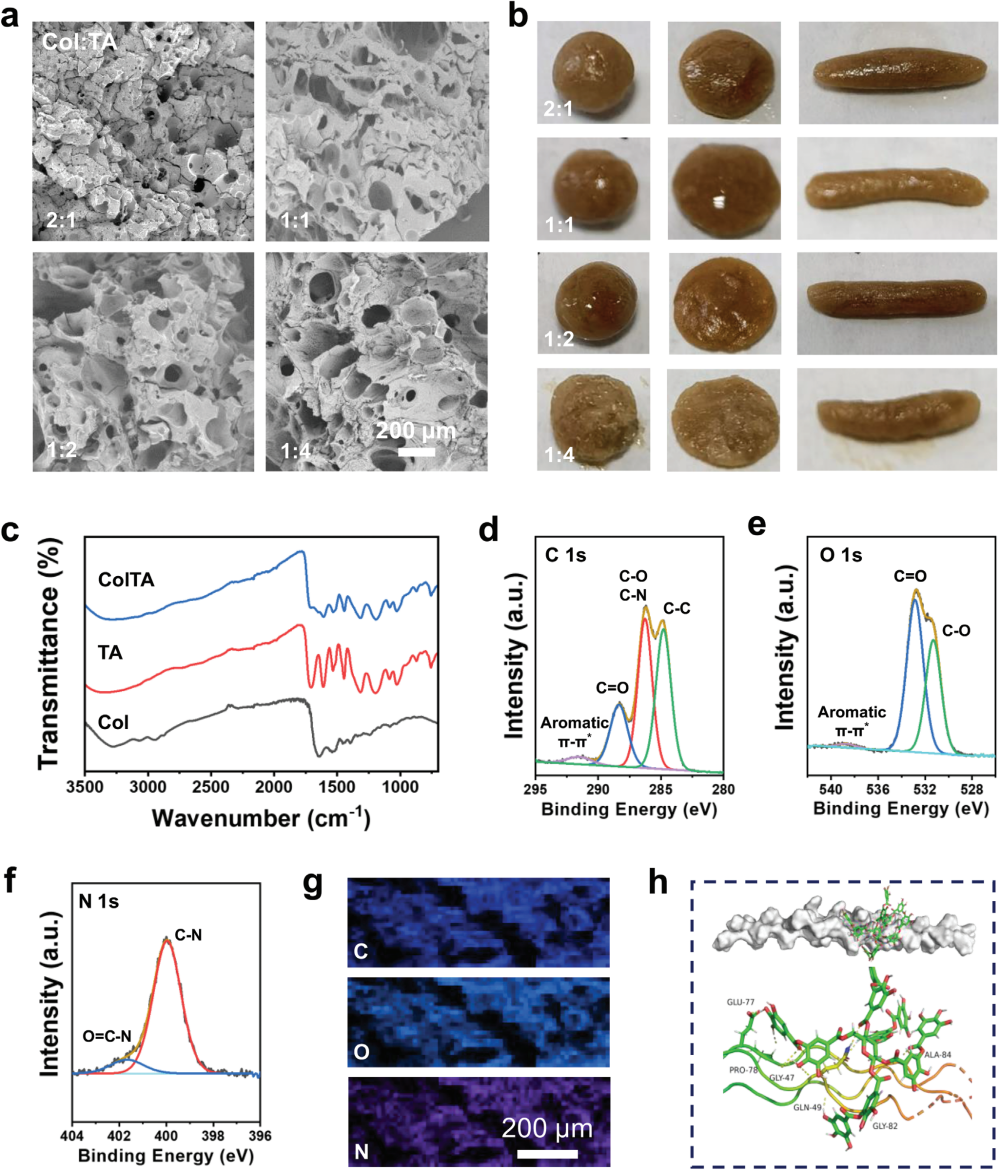
Characterizations of the ColTA adhesives. a) SEM images and b) photographs of the ColTA (water) adhesive at different Col:TA ratios (Col:TA = 2:1, 1:1, 1:2, and 1:4). c) FTIR spectra of Col, TA, and ColTA (water) adhesive. d,e,f) High‐resolution spectra of C 1s, O 1s, and N 1s for the ColTA (water) adhesive. All XPS spectra are plotted after shifting C1s to 284.8 eV. g) The element mapping of the ColTA (water) adhesive. h) The snapshot of ColTA binding posture (Col‐II: white shows the protein surface, TA: green stick), and the visualization of H‐bonds (yellow dotted lines) between Col‐II (irisated ribbon) and TA (green stick) obtained after molecular docking.

The ColTA adhesive was instantaneously formed after simply mixing ColTA powder with water by ultrafast gelation. The micro‐morphology of ColTA (water) adhesive at different Col:TA ratios was observed by scanning electron microscope (SEM) (**Figure** **2**a). All of the adhesives displayed similar 3D porous networks due to the loss of water during the freeze‐drying process. The pore size of the adhesives increased gradually as the Col:TA ratio decreased, suggesting the critical effect of the Col:TA ratio on the microstructure of the adhesive. In addition, ColTA adhesives exhibited excellent plasticity, allowing them to be molded into various shapes including spheres, cubes, and cylinders (Figure 2b). Therefore, the ColTA adhesive was suitable for sealing wounds with irregular shapes. As shown in Figure S1a (Supporting Information), the ColTA adhesive can effectively and rapidly repair a broken water‐filled bottle (Video S1, Supporting Information). Additionally, the adhesive‐repaired area exhibits remarkable stability and cannot be damaged by finger pressure alone, requiring the use of tweezers to disrupt it (Figure S1b and Video S2, Supporting Information). Besides, the pressure test conducted using a water‐filled balloon further demonstrates the exceptional sealing ability of the ColTA adhesive (Figure S1c and Video S3, Supporting Information). Consequently, taking advantage of the excellent adhesion performance, the ColTA adhesive exhibited excellent sealing ability.

The function groups of the ColTA adhesive at the Col:TA ratio of 1:1 were characterized by Fourier transform infrared (FTIR) spectra (Figure 2c). For Col, the absorption peaks at 1637.2, 1541.2, and 1243.8 cm^−1^ were assigned to amide I (C═O stretching), amide II (N─H bending), and amide III (C─N and N─H stretching), respectively (Figure S2a, Supporting Information).^[^ ^21^, ^22^, ^23^ ^]^ In the FTIR spectra of TA (Figure S2b, Supporting Information), several peaks presenting at 1705.9, 1610.3, 1533.5, and 1445.8 cm^−1^ were the characteristics of aromatic compounds.^[^ ^24^, ^25^ ^]^ The presence of absorption peaks at 1196.9, 1086.4, 1027.4, 871.4, and 756.8 cm^−1^ were caused by substituted benzene rings.^[^ ^24^, ^25^ ^]^ The characteristic peaks of Col and TA present in the FTIR spectra of ColTA adhesive (Figure S2c, Supporting Information) due to the physical crosslinking of Col chains with TA. The FTIR spectra of ColTA did not reveal any new characteristic peaks, indicating that the crosslinking between Col and TA was attributed to hydrogen bonding rather than chemical bonding.

X‐ray photoelectron spectroscopy (XPS) has been used to examine the elemental composition of the ColTA (water) adhesive. For Col, the typical peaks of C 1s, O 1s, and N 1s were observed (Figure S3, Supporting Information). The high‐resolution spectra of C 1s can be decomposed into three peaks including the C─C peak at 284.8 eV, C─O/C─N peak at 286.0 eV, and C═O peak at 287.8 eV.^[^ ^26^, ^27^ ^]^ In the high‐resolution spectra of O 1s, the peak at 530.8 eV was assigned to C═O, and the peak at 532.0 eV was caused by C─O.^[^ ^26^, ^27^ ^]^ Besides, the deconvolution of the N 1s peak included the C─N peak at 399.6 eV and the O═C─N peak at 401.2 eV.^[^ ^26^ ^]^ Figure S4 (Supporting Information) shows the high‐resolution spectra of C 1s and O 1s for TA. Four characteristic peaks including the C─C peak (284.8 eV), C─O/C─N peak (286.4 eV), C═O peak (288.9 eV), and aromatic π–π^*^ peak (291.7 eV) present in C 1s spectra.^[^ ^28^, ^29^, ^30^ ^]^ For O 1s, the peaks at 531.6, 533.1, and 539.2 eV were severally assigned to C═O, C─O, and aromatic π–π*.^[^ ^28^, ^29^, ^30^ ^]^ The high‐resolution spectra of C 1s, O 1s and N 1s for the ColTA adhesive were shown in Figure 2d–f. The deconvolution of C 1s peak included the C─C peak at 284.8 eV, C─O/C─N peak at 286.3 eV, C═O peak at 288.4 eV, and aromatic π–π* peak at 291.5 eV.^[^ ^26^, ^27^, ^28^, ^29^, ^30^ ^]^ Besides, the high‐resolution spectra of O 1s can be decomposed into two peaks including C═O peak (531.3 eV) and C─O (532.9 eV), and the peak at 538.7 eV was caused by aromatic π–π*.^[^ ^26^, ^27^, ^28^, ^29^, ^30^ ^]^ For the N 1s spectra, the peak at 399.9 eV was assigned to C─N, and the peak at 401.7 eV was caused by O═C─N.^[^ ^26^ ^]^ XPS results revealed no new peak in the ColTA (water) spectra, suggesting that the crosslinking between Col and TA did not result in the formation of new chemical bonds. Further, the elements of C, O, and N had a consistent distribution (Figure 2g), confirming that the adhesive is homogenous.

The cumulative release rate of TA from ColTA in leachate was detected by measuring the absorbance at 280 nm wavelength. The result showed that the cumulative release rate of TA was gradually increased to ≈12% (w/w%) within 7 days, and then slowed down. At 13 days, the release rate was ≈16% (Figure S5, Supporting Information). Therefore, ColTA adhesive showed the sustained release capacity of TA, thereby exerting various biological functions. In vitro degradation experiments of the materials indicated that the degradation ratio of ColTA adhesive in PBS (without degrading enzyme) was gradually elevated, and at 15 days, the degradation rate increased to nearly 30% (Figure S6, Supporting Information). With the gradual release of TA, ColTA adhesive might degrade slowly.

To confirm the interaction between Col and TA, molecular dynamics simulation (MDS) was performed to assess the stability and kinetic characteristics of the ColTA complex in an aqueous solution. The fluctuation of root mean square deviation (RMSD), an index to evaluate the stability of the system, was small during the simulation, meaning that the system was basically in a stable state (Figure S7a, Supporting Information). The radius of gyration (*R*_g_) was used to estimate the tightness of the system structure, and this index was relatively stable, indicating that the Col chains did not change significantly in the process of simulation (Figure S7b, Supporting Information). Compared with Col group, the protein solvent‐accessibility surface area (SASA) of the ColTA complex decreased slightly (Figure S8a, Supporting Information), attributing to the connection between TA and Col. The number of hydrogen bonds between Col and water in aqueous solution (Figure S8b, Supporting Information) for Col group and ColTA group were calculated respectively. The results showed that the hydrogen bonds between the Col and water were reduced in the presence of TA due to the interaction between Col and TA, which was coincident with the result of SASA (Figure S8a, Supporting Information, in Therapeutic Enzymes: Function and Clinical Implications). The snapshot of the ColTA binding posture showed that there was a long, narrow pocket in the middle of the Col protein, and the TA molecule was located in the middle of the pocket. The results of molecular docking showed that TA combined outside the three ribbon zones (Figure 2h). Further, Pro‐78, Gln‐49, Gly‐47, Leu‐80, Gln‐81, and Ala‐84 of Col can form 0.273, 0.329, 0.274, 0.286, 0.264, and 0.296 nm hydrogen bonds with TA molecule, respectively, surrounded with other hydrophobic amino acids, such as Pro‐20 and Pro‐83 (Figure S9, Supporting Information). Meanwhile, based on the results of binding free energy, the total free energy was ≈−40 kcal mol^−1^, demonstrating that Col and TA had the preferred tendency to combine in chemical kinetics (Figure S10a, Supporting Information). In terms of binding sites, TA molecules were the most important binding stabilizers, confirming the major contribution of TA to the stability of ColTA (Figure S10b, Supporting Information). Therefore, in the presence of an aqueous solution, Col chains were immediately linked with TA through the hydrogen bonds, leading to the ultrafast in situ formation of ColTA self‐gelled adhesive. Meanwhile, the exposed phenol hydroxyls of TA can endow the ColTA adhesive with adhesion property through the formatted physical bonds at the surface of substrates.

### 2.2 Adhesion Property of the ColTA Adhesive

**Figure 3:**
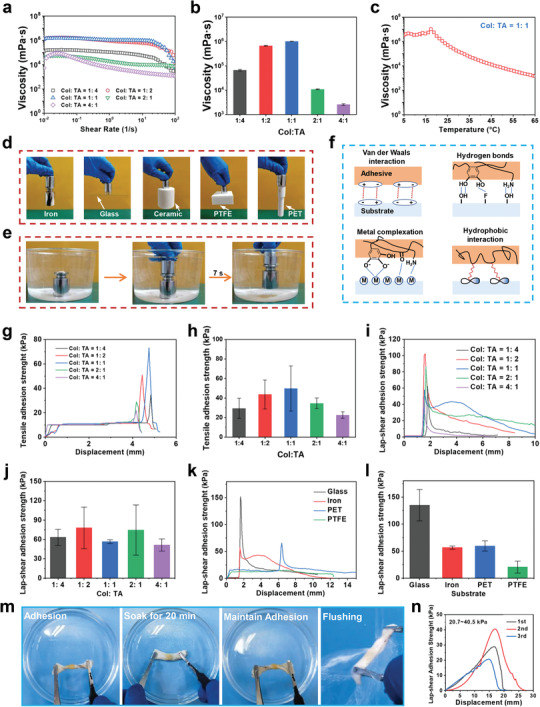
Rheology analysis and adhesion performance of the ColTA (water) adhesives. a) The viscosity curves of the ColTA adhesives on shear rate sweep (10^−2^–10^2^ S^−1^). b) The measured viscosities of the ColTA adhesives at the shear rate of 5–10 S^−1^. c) The viscosity of the ColTA adhesive is a function of temperature. d) The adhesive shows excellent adhesion with various substrates including iron, glass, ceramics, PTFE, and PET. e) The adhesive can instantly and firmly adhere to two steel weights underwater. f) Schematic illustration of the interactions at the interface including van der Waals interaction, hydrogen bonds, metal complexation, and hydrophobic interaction. g) Tensile adhesion curves and h) strength of the adhesives at different Col:TA ratios on iron substrates. i) Lap‐shear adhesion curves and j) strength of the adhesives at different Col:TA ratios on iron substrates. k) Lap‐shear adhesion curves and l) strength of the adhesive (Col:TA = 1:1) on different substrates including iron, glass, PET, and PTFE. m) Stability of the adhesive on rat skin in aqueous solutions and n) related adhesion curves.

The viscosities of adhesives significantly affected their adhesion performance. Therefore, viscosity curves of the ColTA adhesives on shear rate sweep were analyzed to investigate the viscosity properties (**Figure** **3**a). The viscosities of all samples decreased with increasing shear rate, exhibiting a clear shear thinning behavior. Such a decrease in viscosity was mainly caused by the orientation and disentanglement of the adhesive polymers as the shear rate increased.^[^ ^31^, ^32^, ^33^ ^]^ To better clarify the effect of the Col:TA ratio on viscosity, the viscosities of the ColTA adhesives at the shear rate of 5–10 S^−1^ were plotted in Figure 3b. It can be observed that the viscosity first increased gradually and then decreased dramatically as the Col:TA ratio increased. The ColTA adhesive at Col:TA = 1:1 exhibited the highest viscosity among the testing samples. In addition, the viscosity of the adhesive was a function of temperature. As shown in Figure 3c, the viscosity remained unchanged when the temperature increased from 5 to 17.6 °C. However, the viscosity gradually decreased as the temperature further increased from 17.6 to 65 °C, meaning that the temperature harmed the mechanical strength of the ColTA adhesive. Meanwhile, the ColTA adhesive showed excellent adhesiveness to various materials such as iron, glass, ceramic, polytetrafluoroethylene (PTFE), and polyethylene terephthalate (PET) (Figure 3d). In addition, the adhesive can be painted on the substrate and instantly adhere to two steel weights underwater firmly (Figure 3e). The adhesiveness of the ColTA adhesive was mainly attributed to the non‐covalent interactions at the interface. As shown in Figure 3f, those non‐covalent interactions included hydrogen bonds, hydrophobic interaction, metal complexation, and van der Waals interaction.^[^ ^34^, ^35^, ^36^, ^37^ ^]^ The ColTA adhesive can form hydrogen bonds through ─NH_2_, ─OH, ─C═O, and ─COOH function groups with ‐OH groups (or some other function groups containing N, O, and F) on the polymer substrate surface.^[^ ^34^, ^35^, ^38^ ^]^ For metal materials, metal complexation can be formed at the interface.^[^ ^34^, ^35^ ^]^ In addition, hydrophobic interaction and van der Waals interaction also contributed to the adhesion.

To quantitatively investigate the adhesion properties of the ColTA adhesive, tensile adhesion tests were conducted, and adhesive failure (viz., the adhesion failure occurs at the interface) was observed (Figure S11, Supporting Information). The typical tensile adhesion curves of the ColTA adhesive on iron substrates at different Col:TA ratios (1:4, 1:2, 1:1, 2:1, 4:1) were recorded, and the measured tensile adhesion strength was a function of Col:TA ratio (Figure 3g,h). The tensile adhesion strength increased gradually from 29.4 ± 10.4 kPa to 49.8 ± 23.2 kPa as the Col:TA ratio increased from 1:4 to 1:1. However, the tensile adhesion strength started to decrease as the Col:TA ratio further increased from 1:1 to 4:1. These results demonstrated that ColTA adhesive at the Col:TA ratio of 1:1 exhibited the highest tensile adhesion strength among the testing samples.

In addition, lap‐shear adhesion tests were also conducted to investigate the adhesion property under shear load (Figure S12, Supporting Information). The typical lap‐shear adhesion curves of the ColTA adhesive at different Col:TA ratios (1: 4, 1:2, 1:1, 2:1, 4:1) are shown in Figure 3i. Results showed that the Col:TA ratio had little effect on the lap‐shear adhesion strength (Figure 3j). The adhesion strength was primarily determined by the mechanical strength of adhesives and surface interaction at the interface. The result suggested that the variation in mechanical strength caused by changing the Col:TA ratio was insufficient to cause a change in lap‐shear adhesion strength. Compared to the tensile adhesion strength under vertical load, the lap‐shear adhesion strength under shear load was more stable and less susceptible to the Col:TA ratio. Further, the lap‐shear adhesion curves of the ColTA adhesive on typical substrates including glass, iron, PET, and PTFE were measured (Figure 3k). The substrates significantly determined the lap‐shear adhesion strength, and the adhesion strength of the adhesive on glass was the highest among the tested substrates (135.0 ± 29.0 kPa) (Figure 3l), mainly attributing to hydrogen bonds. The adhesion strength of the ColTA adhesive on PTFE was only 20.6 ± 11.0 kPa, which was much lower than that on other substrates due to its low surface energy (≈19 mJ m^−2^).^[^ ^39^ ^]^ Besides, the adhesion strength of the ColTA adhesive was also a function of the temperature (Figure S13a, Supporting Information). The lap‐shear adhesion strength decreased from 56.7 ± 2.9 kPa to 15.7 ± 5.9 kPa as the temperature increased from 25 to 55 °C (Figure S13b, Supporting Information). The decrease in adhesion strength was mainly caused by the loss in viscosity at high temperatures (Figure 3c). Further, increasing the temperature can also diminish the interaction at the adhesive‐substrate interface,^[^ ^40^, ^41^, ^42^ ^]^ which contributes to decreasing adhesion. In addition, rat skin was used as a model to confirm the adhesion performance between the ColTA adhesive and tissue. As shown in Figure 3m, the ColTA adhesive can tightly adhere to wet skin even after being immersed in water for 20 min. In addition, the adhesive can maintain adhesion even after water flushing. Lap‐shear adhesion tests showed that the adhesion strength of the ColTA adhesive on the rat skin was 20.7–40.5 kPa (Figure 3n). In addition, the measured lap‐shear adhesion strength of the ColTA adhesive on porcine skin is 29.2–45.8 kPa (Figure S14, Supporting Information), which is comparable with that on the rat skin. These results demonstrated the excellent adhesion performance of the ColTA adhesive. Further, the adhesives based on other polyphenols such as epicatechin (EG) and gallic acid (GA) also were prepared. It was clear that the ColTA adhesive possessed the best performance due to the highest number of phenolic hydroxyl groups of TA (Figure S15, Supporting Information).

### 2.3 Blood‐Enhanced Adhesion Properties of the ColTA Adhesive

**Figure 4:**
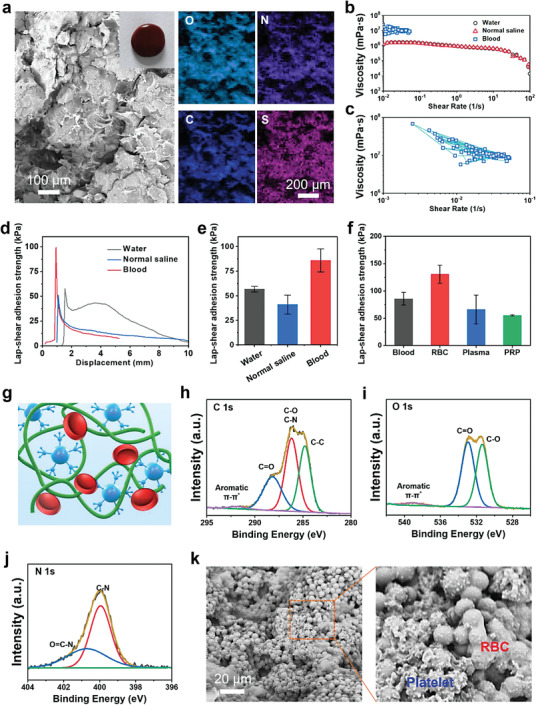
Rheology analysis and adhesion performance of the ColTA (blood) adhesives. a) SEM image and element mapping of the ColTA (blood) adhesive. b) The viscosity curves of the ColTA adhesives with different solutions (water, normal saline, and blood). c) Detailed viscosity curve of the ColTA (blood) adhesive. d) Lap‐shear adhesion curves and e) strength of the ColTA adhesives with different solutions (water, normal saline, and blood) on iron substrates. f) The lap‐shear adhesion strength of the ColTA (blood), ColTA (RBC), ColTA (plasma), and ColTA (PRP) adhesives on iron substrates. g) Schematic illustration of the blood‐enhanced adhesion mechanism. High‐resolution spectra of h) C 1s, i) O 1s, and j) N 1s for the ColTA (blood) adhesive. All XPS spectra are plotted after shifting C1s to 284.8 eV. k) SEM image of blood cells on the surface of the ColTA adhesive.

During the hemostasis process, blood rather than water induced the self‐gel of ColTA adhesive. Therefore, the ColTA (blood) adhesive was further characterized. Four elements including O, C, N, and S were observed and possessed consistent distribution based on SEM image and element mapping of the ColTA (blood) adhesive (**Figure** **4**a). The viscosity curves of the ColTA adhesives with different solutions (water, normal saline, and blood) were compared (Figure 4b). It can be observed that the viscosity of the ColTA (blood) adhesive was much larger than that of the ColTA (water) and ColTA (normal saline) adhesives, and the viscosity of the ColTA (blood) adhesive exceeded the detection limit of the rheometer when the shear rate was >0.05 s^−1^ (Figure 4c). The typical lap‐shear adhesion curves of the ColTA adhesives with different solutions (water, normal saline, and blood) showed that the solution significantly affected the measured lap‐shear adhesion strength (Figure 4d,e). The adhesion strength of the ColTA (blood) adhesive reached 85.9 ± 11.6 kPa, which was much larger than that of the ColTA (water) adhesive (56.7 ± 2.9 kPa) and ColTA (normal saline) adhesive (41.0 ± 9.7 kPa). The high adhesion strength of the ColTA (blood) adhesive was mainly attributed to its large viscosity (Figure 4b,c). To further confirm which component in the blood plays the main role, RBCs, blood plasma, and platelet‐rich plasma (PRP) were used to mix with ColTA powder for adhesion tests. As shown in Figure 4f, the adhesion strength of the ColTA (RBC) adhesive was significantly larger than that of the ColTA (blood), ColTA (plasma), and ColTA (PRP) adhesives, suggesting the dominant rule of RBCs in the blood‐enhanced adhesion performance. The phenol hydroxyl groups and amino groups on the ColTA adhesive polymers can absorb RBCs (especially membrane protein) through electrostatic interactions,^[^ ^6^, ^43^, ^44^, ^45^, ^46^ ^]^ subsequently leading to the aggregation of the polymer chains with RBCs. During the lap‐shear teats, the RBCs trapped and coagulated within the adhesive polymers can resist deformation (Figure 4g), thereby contributing to higher adhesion strength.

Meanwhile, the elemental composition of the ColTA (blood) adhesive was further examined by XPS spectra. As shown in Figure 4h, the high‐resolution spectra of C 1s can be decomposed into four peaks including C─C peak (284.8 eV), C─O/C─N (286.2 eV), C═O (288.2 eV), and aromatic π–π^*^ peak (291.7 eV).^[^ ^26^, ^27^, ^28^, ^29^, ^30^ ^]^ The deconvolution of O 1s peak included the C─O peak at 531.4 eV, the C═O peak at 532.9 eV, and the aromatic π–π^*^ peak at 539.1 eV (Figure 4i).^[^ ^26^, ^27^, ^28^, ^29^, ^30^ ^]^ In Figure 4j, the peak at 399.95 eV was assigned to C─N, and the peak at 400.7 eV was caused by O═C─N.^[^ ^26^ ^]^ In addition, very weak peaks can be observed in S 2p spectra, which was caused by the anticoagulant drugs in the collected blood (Figure S16, Supporting Information). In addition, the capability of the in situ formed ColTA adhesive to aggregate blood cells was examined by dropping the blood on the adhesive. The obvious aggregation and adhesion of RBCs and platelets on the ColTA adhesive surface appeared (Figure 4k).^[^ ^6^ ^]^ Therefore, ColTA adhesive powder can rapidly absorb blood to instantaneously form in situ adhesive for enhanced hemostasis when deposited on the bleed sites.

### 2.4 Cytocompatibility and Antibacterial Activity of ColTA Powder

**Figure 5:**
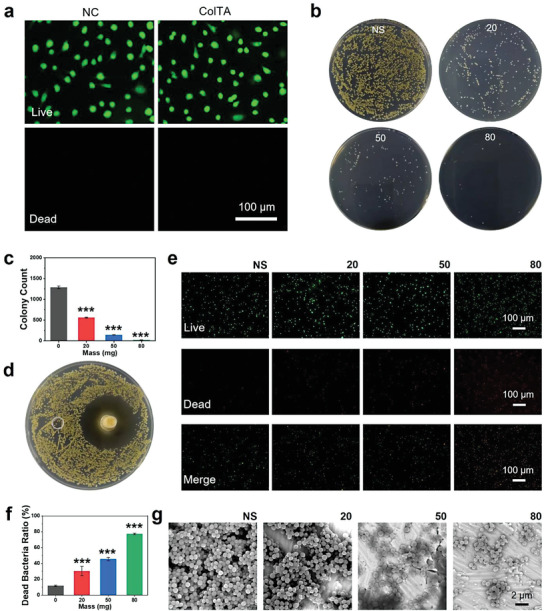
Cytocompatibility and antibacterial activity of ColTA powder. a) Live and dead cell staining of HUVECs treated with material leach liquors for 48 h. b) Photographs of bacterial colonies from *S. aureus* incubated with various concentrations of material leach liquors. c) Counting for the number of *S. aureus* colonies. d) Photograph of bacterial inhibition ring after incubation with the self‐gelling ColTA hydrogel. e) Live and dead bacteria staining of the *S. aureus* incubated with various concentrations of material leach liquors. f) Counting for the ratios of dead bacteria. g) Bacterial biofilms formed by *S. aureus* incubated with various concentrations of material leach liquors on titanium surface. ^***^ *p* < 0.001 compared with the group without materials.

Col‐II is an important structural protein in the extracellular matrix and has been broadly used in biomedical applications.^[^ ^47^ ^]^ TA with good bioactivities is a natural polyphenol derived from the bark or fruit of plants.^[^ ^20^ ^]^ Considering the nature of the above raw materials, the self‐gelling ColTA adhesive should be highly biocompatible. The results of live and dead cell staining presented a similar proportion of living cells (green) by contrast with the control group after cultivation with the ColTA material leach liquor for 48 h (**Figure** **5**a). Cell counting kit‐8 (CCK‐8) assay also indicated that the relative cell viability of the ColTA group was >80%, which conformed to the ISO10993‐5 standard of biomaterial cytocompatibility (Figure S17a, Supporting Information). Therefore, the ColTA powder and its self‐assembled adhesive were cytocompatible.

*Staphylococcus aureus (S. aureus)* were incubated with the material leach liquors and coated to the agar plates to appraise the antibacterial activity of ColTA powder. The number of bacterial colonies significantly decreased with the concentration of material leach liquors (Figure 5b,c). A bacterial inhibition ring test was performed to evaluate the antibacterial capacity of the self‐gelling ColTA adhesive. *S. aureus* suspension was spread to the agar plate, and then the container containing hydrogels was placed on the agar plate. Compared with the negative control group (normal saline), the bacteriostasis ring caused by ColTA adhesive was obvious, and the diameters of the rings were ≈43 mm (Figure 5d; Figure S17b, Supporting Information). Furthermore, *S. aureus* were stained with a Live/Dead bacteria staining kit. The results showed that the ratio of dead bacteria increased significantly in leach liquor groups (Figure 5e,f). Ulteriorly, the bacterial biofilms formed by *S. aureus* after incubation with material leach liquors were investigated by SEM. Compared to the normal saline group, the biofilms in leach liquor groups were fragmentary, and the corresponding density of bacteria notably decreased (Figure 5g). The above results demonstrated that the ColTA powder was highly capable of sterilizing *S. aureus*, which should be owing to the antibacterial property of TA.^[^ ^20^ ^]^ Fibrin glue as a commercial hemostatic product with biological activities has been widely used in surgical operations for hemostasis and tissue adhesion applications. However, the fibrin glue is a good medium for microbial growth, increasing the risk of postoperative infection. We also demonstrated that fiber glue had no antibacterial effect, and when the usage is a little high, it would promote bacterial proliferation (Figure S18, Supporting Information).

### 2.5 Hemocompatibility and Hemostatic Effect of ColTA Powder

**Figure 6:**
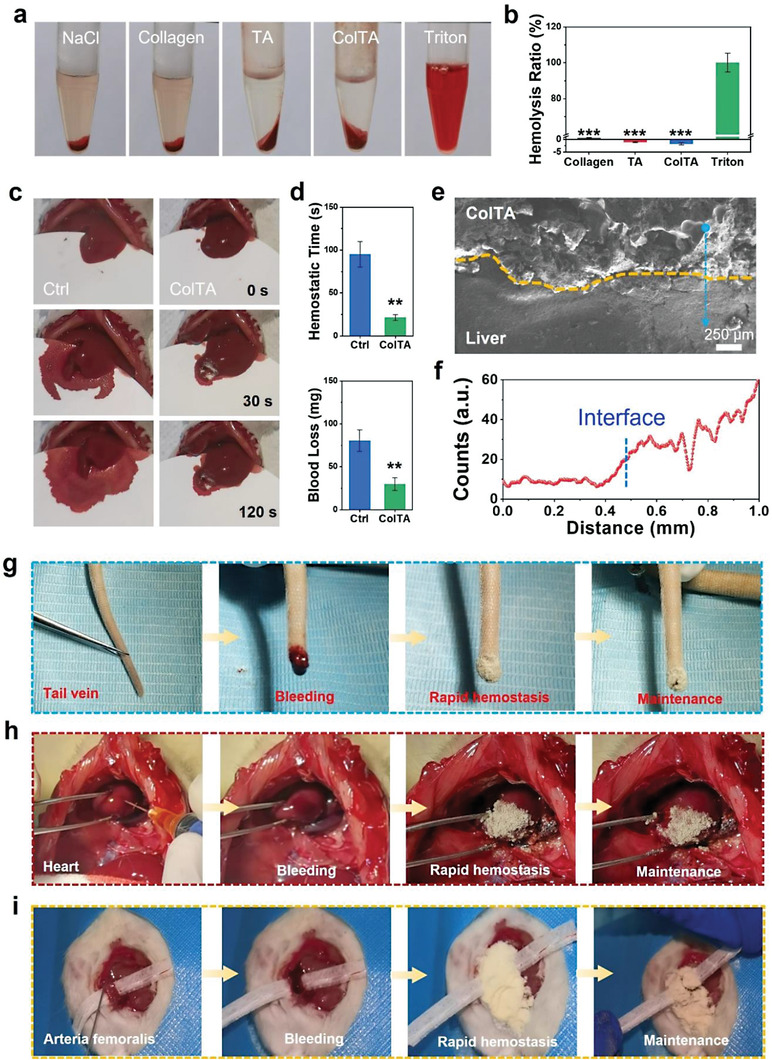
Hemocompatibility and hemostatic effect in vivo of ColTA powder. a,b) Hemolytic test of the Col‐II, TA, and ColTA powder, and the hemolysis ratio. c) Bleeding of the liver and hemostasis with ColTA powder in rat models. d) The quantification for bleeding time and blood loss of liver e) Image of EPMA for liver‐ColTA interface (dotted yellow line). The blue arrow represents the direction and position of the line scan. f) The analysis of sulfur element distribution with line scan mode by EPMA. g–i) Bleeding of the tail vein, heart, and arteria femoralis, and hemostasis with ColTA powder in rat models. ^***^ *p* < 0.001, ^**^ *p* < 0.01 compared with group without materials.

Excellent hemocompatibility is an essential property for hemostatic powders. The ColTA powder will come into contact with the bleeding wounds and is not expected to elicit a hemolytic reaction. Therefore, the in vitro hemolysis tests were performed to verify the hemocompatibility of the ColTA powder. Normal saline was set as the negative control group, and 0.1% Triton‐X 100 was set as the positive control group. The centrifugal supernatant of the ColTA group was the most transparent and clearest among all groups, and the TA group came second, which might be owing to the powerful affinity of TA with biological macromolecules and RBCs (**Figure** **6**a).^[^ ^48^ ^]^ Synchronously, the hemolysis rate calculated from the OD_570_ of the supernatants in the ColTA group and TA group was significantly lower than that of the NaCl group (Figure 6b, *p* < 0.001), which suggested that TA might endow ColTA powder with the capacity of adsorbing RBCs once contacting the bleeding wounds.

The in vitro blood clotting indexes (BCI) were detected by measuring the absorbance at 540 nm (Figure S19, Supporting Information). Combining the literature and in vitro blood clotting time, the amount of hemostatic material used is ≈20 mg of ColTA powder for 50 µL blood. The results showed that the clotting time and blood clotting indexes of the ColTA group were significantly decreased in comparison with the control group, which might be owing to the enhanced adhesion performance in the presence of blood due to the electrostatic interactions between ColTA and red blood cells.

The hepatic hemorrhage model in rats was used to assess the hemostatic effect in vivo of ColTA powder (Figure 6c). The liver was cut with a 0.5 cm long wound, where much blood gushed out, and continued for ≈100 s with 90 mg of blood loss. The bleeding was stopped within 20 s after applying ColTA powder to the wounds, and the blood loss was significantly reduced to 30 mg (Figure 6d). At the liver‐ColTA interface, ColTA powder can self‐gelatinize upon contact with blood, and adhere to the wounded liver incisions, which can form a physical barrier to seal the bleeding wound section (Figure 6e). Electro‐probe microanalyzer (EPMA) analysis with line scan mode indicated a gradual increase in the sulfur content from ColTA to the liver, without interruption at the interface (Figure 6f), suggesting a continuous liver‐ColTA interface with ColTA firmly adhering to the liver incision.

Meanwhile, tail vein, heart, and arteria femoralis bleeding models in rats were used to evaluate the hemostatic effect of ColTA powder for irregularly shaped and incompressible wounds. The tail of the rat was cut off to induce the caudal vein bleeding, and ColTA powder could absorb blood quickly, thereby concentrating the coagulation factors and stopping bleeding within 20 s (Figure 6g). The heart and arteria femoralis of rats were exposed and punctured to bring about acute bleeding (Figure 6h,i). The application of ColTA powder could efficiently cease bleeding and seal the bleeding sites within 20 s. After hemostasis, these incompressible wounds were not bleeding again, even if stretched (Figure 6i). Therefore, the above results showed that ColTA powder could effectively exercise the function of hemostasis and avoid secondary bleeding.

### 2.6 Immunomodulatory Effect of ColTA Powder on RAW 264.7 Macrophages

**Figure 7:**
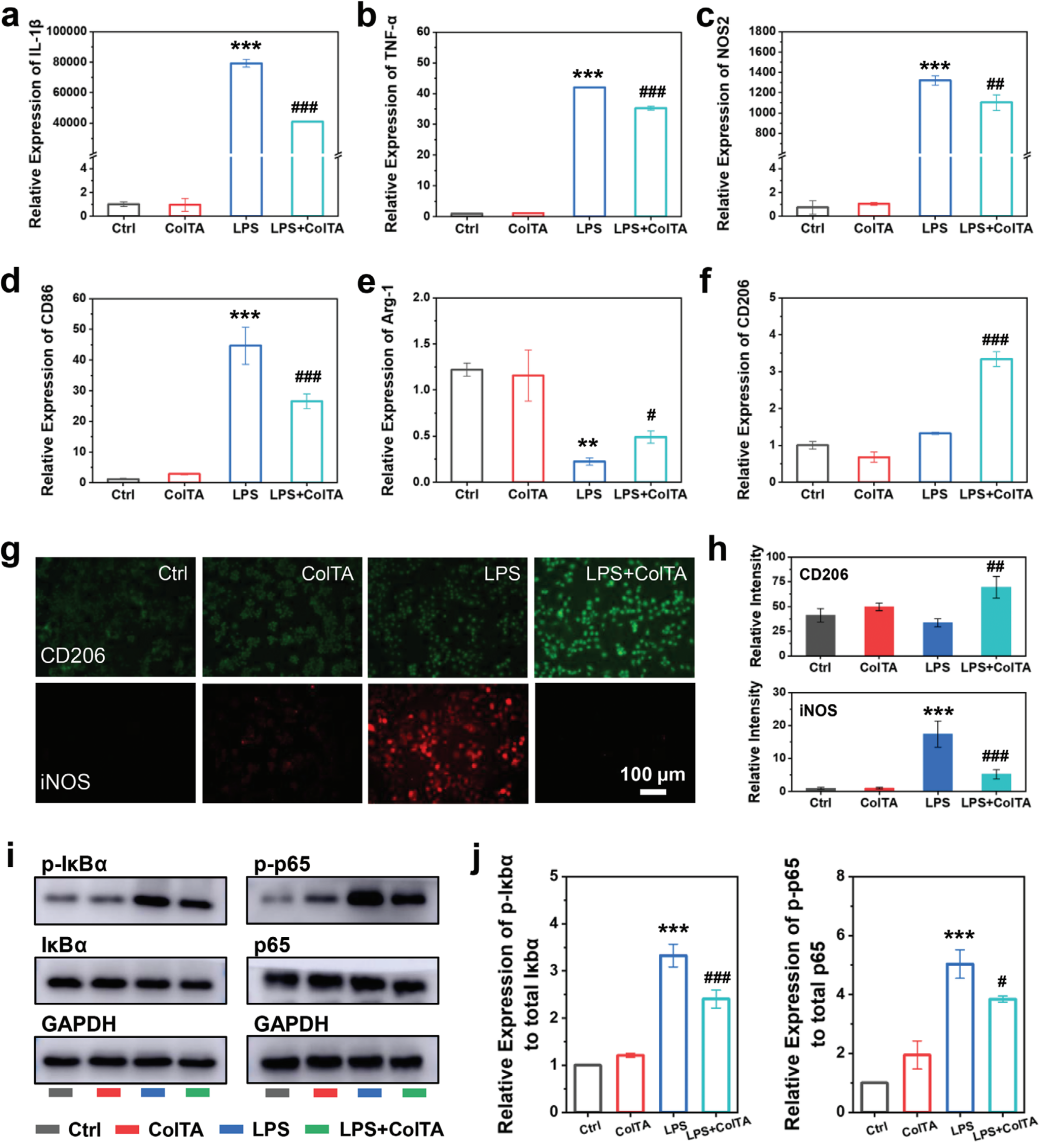
Immunomodulatory effect of ColTA powder on RAW 264.7 macrophages. a–f) The relative expression level of IL‐1β, TNF‐α, NOS‐2, CD86, Arg‐1, and CD206. g) The immunofluorescent staining for CD206 (green) and iNOS (red) in RAW 264.7 cells. h) The corresponding quantitative analysis for the fluorescence intensity of CD206 and iNOS. i) The activation of the NF‐κb signaling pathway including the phosphorylation level of Iκbα and p65. The protein expression levels of p‐Iκbα, p‐p65, Iκbα, p65, and GAPDH were detected by western blot. j) The corresponding quantitative analysis for the phosphorylation of Iκbα and p65. ^***^ *p* < 0.001, ^**^ *p* < 0.01 compared with control group (Ctrl); ^###^ *p* < 0.001, ^##^ *p* < 0.01, ^#^ *p* < 0.05 compared with LPS group.

Injury bleeding is often accompanied by wound immuno‐inflammatory response and tissue damage. Therefore, ideal hemostatic materials should also contribute to immunoregulation and wound healing. The immuno‐inflammatory response is the indispensable phase closely related to the repair of damaged tissue. Macrophages, owing to their central modulatory roles in the process of tissue healing, have served as the crucial target of immunomodulatory and wound healing strategies.^[^ ^49^ ^]^ As is well‐known, M0 macrophages would polarize into proinflammatory/classically activated M1 phenotype, and anti‐inflammatory/alternatively activated M2 phenotype induced by different immune‐inflammatory cues. In the early stage of tissue injury, activated M1 macrophages contribute to the essential inflammatory response by secreting inflammatory cytokines (e.g., tumor necrosis factor (TNF)‐α and interleukin (IL)‐1β), and produce high levels of inducible nitric oxide synthase (iNOS) and reactive oxygen species (ROS), which can eradicate pathogens and debride the impaired tissue. However, the persistent activation of M1 macrophages would bring about prolonged chronic inflammation, thereby impeding wound healing.^[^ ^50^ ^]^ Therefore, mitigation of the excessive M1 response and skewing polarization to the M2 phenotype has become an acknowledged immunoregulatory strategy. The gene expression levels of inflammatory cytokines (IL‐1β and TNF‐α), M1 phenotype markers (CD86 and nitric oxide synthase (NOS)2), and M2 phenotype markers (CD206 and arginine (Arg)‐1) were detected by quantitative real‐time polymerase chain reaction (qRT‐PCR). The expression of inflammatory cytokines (IL‐1β and TNF‐α), and M1 phenotype markers (NOS2 and CD86) were significantly up‐regulated by lipopolysaccharide (LPS) stimulation, which can be efficiently restrained with ColTA material leach liquor (**Figure** **7**a–d). Meanwhile, compared with the LPS group, the expression of M2 phenotype markers (Arg‐1 and CD206) was augmented after ColTA leach liquor treatment (Figure 7e,f). Immunofluorescent staining was performed to further evaluate the expression of iNOS and CD206 in macrophages treated with or without ColTA leach liquor. The results showed that LPS‐induced iNOS expression was inhibited by ColTA leach liquor, and conversely, the expression of CD206 was increased in the ColTA‐treated group compared to the LPS group, which was consistent with the results of qRT‐PCR (Figure 7g,h).

The canonical nuclear fator‐kappa B (NF‐κB) pathway, that is the activation of p65/p50 heterodimer triggered by stimulus such as LPS, has long been considered as a prototypical signaling pathway for proinflammatory reaction and M1 polarization.^[^ ^51^ ^]^ Generally, inhibitors of NF‐κB (IκBs) can sequester NF‐κB p65/p50 heterodimer in the cytoplasm. IκBα is phosphorylated and degraded once stimulated by LPS, liberating the p65/p50 complex to translocate into the nucleus, thereby initiating the expression of inflammatory target genes. In addition, LPS‐stimulated phosphorylation of p65 is indispensable for the transcriptional activation of p65/p50 heterodimer.^[^ ^52^ ^]^ The activation of the NF‐κB pathway was detected by western blot. The results indicated that ColTA can inhibit LPS‐stimulated phosphorylation of IκBα (the best‐characterized member of IκBs) and p65 (Figure 7i,j). Consequently, ColTA can inhibit the excessive immune‐inflammatory response and M1 polarization in LPS‐induced RAW 264.7 macrophages via the NF‐κB pathway. Crucially, ColTA promoted M2 polarization of macrophages in the presence of LPS, which can provide a microenvironment conducive to wound healing.

### 2.7 Pro‐Healing Effects and In Vivo Biosecurity of ColTA Powder on Rat Skin Wound

As mentioned above, in addition to the satisfactory hemostatic function, advantageous pro‐healing effects and immunomodulatory capacities are also critical elements for an ideal hemostatic material. An infectious skin defect model of rats was prepared by making a full‐thickness round skin defect (diameter: 8 mm) with *S. aureus* infection on the back of rats. The biosecurity of ColTA powder was confirmed first. The results of hematoxylin&eosin (H&E) staining for visceral organs showed that there was no significant difference in the organs of the ColTA group in comparison to the control group (Figure S20, Supporting Information). Meanwhile, The blood cell analysis also indicated that the blood cells were within the normal range in the ColTA group (Tables S1 and S2, Supporting Information). Therefore, the self‐gelling ColTA adhesive showed no systemic toxicity.

**Figure 8:**
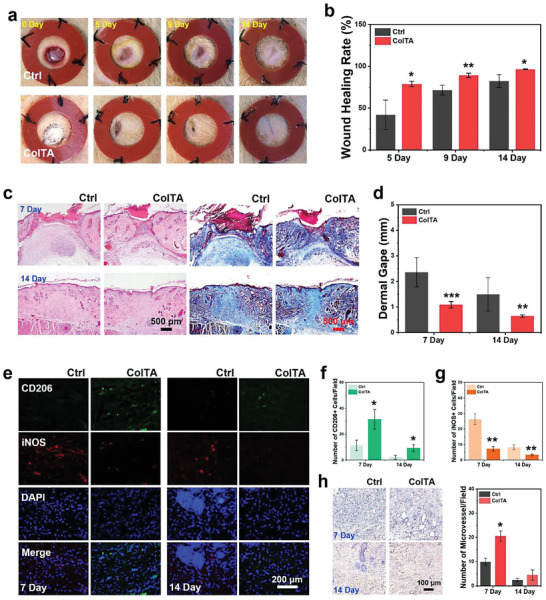
Pro‐healing effects of ColTA powder on rat skin wounds. a) Photographs of infectious skin defect models treated with ColTA powder at days 0, 5, 9, and 14. b) The corresponding wound healing rate of the skin defects. c) H&E staining and Masson staining of the skin defects at days 7 and 14. d) The quantitative analysis for the width of the dermal gape at days 7 and 14. e) The immunofluorescent staining for CD206 and iNOS at days 7 and 14. f) Counting for the number of CD206+ cells. g) Counting for the number of iNOS+ cells. h) Immunohistochemical staining of α‐SMA expression and counting for the number of α‐SMA positive capillaries at days 7 and 14 after treatment. The circular brown staining indicated blood vessels. ^***^ *p* < 0.001, ^**^ *p* < 0.01, and ^*^ *p* < 0.05 compared with control group (Ctrl).

Gross observation showed that the wounds healed better in the ColTA group by contrast with the control group (**Figure** **8**a). The calculated wound healing rate presented results consistent with general observation during the whole healing process (Figure 8b). H&E staining and Masson staining of skin tissues at days 7 and 14 were performed to further evaluate the pro‐healing effect of ColTA powder. The width of dermal gaps in the ColTA group was significantly narrowed compared with the control group (Figure 8c,d). These results illustrated that ColTA powder had pro‐healing effects on rat infectious skin wounds without significant toxic effects on the surrounding skin tissue.

As mentioned above, mitigation of the excessive M1 response and skewing polarization to anti‐inflammatory M2 phenotype are conducive to wound healing. Further, CD206+ and iNOS+ cells in vivo were detected with immunofluorescent double staining for CD206 (green) and iNOS (red) to evaluate the reaction of macrophages to ColTA powder during skin wound healing process (Figure 8e). At day 7, more CD206+ cells were observed in the ColTA group, and the number of iNOS+ cells was significantly reduced in comparison with the control group (Figure 8e–g). At day 14, the number of positive cells overall decreased in all groups by contrast with day 7, whereas the ColTA group still presented more CD206+ cells and fewer iNOS+ cells than the control group (*p* < 0.05). Consequently, ColTA powder can contribute to skewing the polarization of macrophages to M2 phenotype for wound healing promotion, which was consistent with the results of in vitro experiments. Therefore, the pro‐regenerative, anti‐inflammatory, and immunoregulatory properties of ColTA are synergistically devoted to wound healing.

Angiogenesis is an essential process for wound healing since neovascularization can supply oxygen, nutrients, and access to cell circulation needed for tissue healing. α‐smooth muscle actin (α‐SMA) is mainly expressed at the blood vessel wall, and conducive to the formation of vascular smooth muscle cell cytoskeleton.^[^ ^53^ ^]^ The revascularization in skin defects was evaluated with immunohistochemical staining for angiogenesis‐related markers α‐SMA (Figure 8h). The positive brown staining with the typical round or oval structure in the ColTA group was more obvious than that in the control group on day 7. At day 14, the positive staining for α‐SMA decreased significantly, and there was no statistical difference between the two groups. Therefore, ColTA powder can facilitate neovascularization in the early stage of skin wound healing, which can provide adequate blood supply for tissue repair.

## 3 Conclusion

In summary, this study reports a multifunctional and hydro‐sensitive ready‐to‐use ColTA adhesive powder that exhibits rapid hemostasis suitable for various types of bleeding wounds by in situ ultrafast gelation. The ColTA powder can absorb blood quickly and self‐gel to form adhesive without any chemical crosslinking, and the formed adhesive exhibits excellent adhesion performance on various materials through various non‐covalent interactions at the interface. In addition, the self‐gelled ColTA adhesive presents the RBCs‐enhanced adhesion performances by the electrostatic interactions between ColTA adhesive and RBCs, which is conducive to rapid hemostasis and sealing of bleeding sites. The ColTA powder with eligible biocompatibility and hemocompatibility can stop bleeding of incompressible or irregularly shaped wounds in rat hemorrhage models. Furthermore, ColTA adhesive powder also possesses favorable bioactivities of antibiosis, immunoregulation, and wound repair promotion. Consequently, the accessible, low‐cost, and user‐friendly ColTA powder would be a prospective hemostatic material for complex bleeding, and the instantaneous self‐gelling behavior of Col‐II and TA provides a promising strategy for the development of new hemostatic materials.

## 4 Experimental Section

### ColTA Adhesive Preparation

Col‐II and TA were purchased from Shanghai Macklin Biochemical Technology Co., Ltd, China. Wistar rats (male, 8 weeks, Vital River, Beijing, China) were used to collect the whole blood approved by the Medical Ethics Committee of Stomatological Hospital, Shandong University, Jinan, China (Permit Number: No. 20220310) and based on the ethical guidelines of the Care and Use of Laboratory Animals of the Chinese Science and Technology Ministry. RBCs, PRP, and blood plasma were collected by centrifuging the anticoagulated whole blood. Col‐II and TA were mixed in different mass proportions (Col:TA = 4:1, 2:1, 1:1, 1:2, 1:4) to obtain ColTA powder. Subsequently, 300 mg of powder was weighed and mixed with 200 µL of aqueous solutions (water, normal saline, anticoagulated blood, RBCs, PRP, and blood plasma) to obtain the self‐gelling ColTA adhesive, which was referred to as ColTA (water), ColTA (normal saline), ColTA (blood), ColTA (RBC), ColTA (PRP), and ColTA (plasma). Then, the adhesives were freeze‐dried for subsequent material characterizations.

### Characterizations

The surface morphology characteristics of the freeze‐dried adhesives were observed under a Phenom ProX SEM (Phenom, Holland). Chemical composition was evaluated by FTIR (Thermo Scientific, USA). Elemental composition was assessed by XPS (Thermo Scientific, USA). The liver‐material interface elemental analysis was performed by an EPMA (Shimadzu, Japan). A rheometer (MCR 302, Anton Paar, Austria) was used to measure the viscosities of the ColTA adhesives at different Col:TA ratios at 25 °C. In addition, the ColTA adhesive (Col:TA = 1:1) at a temperature range of 5–65 °C was also conducted at 5 s^−1^.

### Molecular Simulation

The structure of type II collagen with (Pro‐Hyp‐Gly)3‐Arg‐Ala‐Gly‐Glu‐Pro‐Gly‐Leu‐Gln‐Gly‐Pro‐Ala‐Gly‐(Pro‐Hyp‐Gly)3 was acquired from RSCB Protein Data Bank (PDB; ID 6JEhttps://doi.org/10.2210/pdb6JEC/pdb). The three helices composed of twelve amino acid residues were the common motif of human type II collagen.^[^ ^54^ ^]^ Molecular docking was performed using Smina software, and the conformation of the docking result was used as the initial conformation. Molecular dynamics simulation was performed with Gromacs 2019.6 procedure and amber14sb protein field. The TIP3P water model was applied to the complex system to establish a water box, and sodium ion was added to the balance system. The steepest descent method was used to minimize the energy of the maximum number of steps (5000 steps). NVT and NPT were used for 30 ps equilibrium simulation, and then 100 ns molecular dynamics simulation was performed at normal temperature and pressure. During MD simulation, hydrogen bonds were constrained with the LINCS algorithm, and the integral step was 2 fs. Particle‐mesh Ewald (PME) method was used to calculate the electrostatic interaction. The non‐bond interaction cutoff value was set to 10 Å. The V‐rescale temperature coupling method was used to control the simulated temperature of 300 K, and the Berendsen method was used to control the pressure of 1 bar. The simulation results were visualized with PyMOL 2.5.1 and LigPlot 2.1.

### Adhesion Tests

Adhesion tests were performed using a universal testing machine (ZLC‐2D, Jinan XLC Testing Machine Co., Ltd, China) equipped with a 500 N load cell at a loading rate of 50 mm min^−1^. Unless otherwise specified, the tests were conducted at 25 °C. In the tensile adhesion tests, the ColTA adhesive was painted on a polished rod, and then another rod with a diameter of 15 mm was pressed against the surface. The lap‐shear adhesion tests of the ColTA adhesive were conducted with a square contact area of 12 mm × 12 mm. The lap‐shear adhesion tests were used to evaluate the adhesion performance of the ColTA adhesive on various substrates including glass, iron, PET, and PTFE. The lap‐shear adhesion tests of the ColTA adhesive on rat skin were conducted to evaluate the tissue adhesiveness. In addition, the adhesion tests at different temperatures (25, 35, 45, and 45 °C) were conducted in a temperature‐controlled chamber (Jinan XLC Testing Machine Co., Ltd, China). Unless otherwise specified, the default substrates used for the adhesion tests were iron substrates.

### In Vitro Releasing of TA from ColTA Adhesive

One milliliter ColTA adhesive was prepared and immersed into 10 mL phosphate buffer saline (PBS) in a centrifugal tube. Then, the centrifugal tubes for different time points were placed in a constant temperature shaker (37 °C, 100 rpm). The liquid supernatants were collected at certain points, and the absorbance values at 280 nm wavelength were measured with an ultraviolet spectrophotometer. The cumulative release rate of TA from ColTA in leachate was obtained by calculating the concentration of TA according to the standard curve.

### In Vitro Degradation of ColTA Adhesive

1 mL ColTA adhesive was prepared and immersed into 10 mL PBS in a centrifugal tube. Then, the centrifugal tubes for different time points were placed in a constant temperature shaker (37 °C, 100 rpm). At different time points, the PBS in the centrifugal tubes was discarded, and the remaining ColTA adhesive was lyophilized and weighed, thereby calculating the degradation ratio of ColTA adhesive in PBS.

### Cell Culture and Cytocompatibility

Human umbilicus vein endothelial cells (HUVECs) were cultured in endothelial cell medium (ECM, Sciencell, USA) containing 1% endothelial cell growth supplement, (ECGS, Sciencell), and 5% Foetal Bovine Serum (FBS, Sciencell). The cells were inoculated to 24 well culture plates. The next day, the medium was replaced with material leach liquors which were prepared by adding 200 mg powder to a centrifuge tube filled with 10 mL ECM placed in a constant temperature oscillator (37 °C, 100 rpm) for 48 h, and then cells were incubated at 37 °C, 5% CO_2_ incubator for 48 h. The cytocompatibility was assessed by CCK‐8 (Dojindo Laboratories, Kumamoto, Japan) assay and Live/Dead cell staining (Solarbio, Beijing, China) by the manufacturer's protocols, respectively.

### Antibacterial Tests

*S. aureus*) were diluted to 1 × 10^5^ CFU mL^−1^ in the material leach liquors, and incubated at 37 °C for 12 h. The material leach liquors were acquired by adding 20, 50, and 80 mg ColTA powder to 1 mL of normal saline in a constant temperature oscillator (37 °C, 100 rpm) for 12 h, respectively. Subsequently, 20 µL of above *S. aureus* suspensions were coated to the agar plates and incubated for 12 h at 37 °C. The bacterial colonies were counted by using Image J 1.44 software (NIH, Bethesda, Maryland, USA). In addition, the above *S. aureus* suspensions were also used for Live/Dead bacteria staining (BestBio, Shanghai, China) to further evaluate antimicrobial activity. The bacteria were also counted with Image J software, and then the ratio of dead bacteria to total bacteria was calculated.

A bacterial inhibition ring test was also performed to confirm the antibacterial ability. Briefly, the self‐gelling ColTA adhesives were injected into a cylindrical container (diameter and height: 4 mm). 50 µL of *S. aureus* suspension (1 × 10^5^ CFU mL^−1^) was spread to the agar plate, and then the container containing ColTA adhesives was placed on the agar plate, and incubated for 12 h at 37 °C. Normal saline was used as the negative control group. The diameters of the bacteriostasis rings were measured with Image J software. Additionally, bacterial biofilms formed by *S. aureus* were also observed by SEM. *S. aureus* were inoculated onto the surface of the titanium sheet. 300 µL of *S. aureus* suspension (1 × 10^8^ CFU mL^−1^) was added into a 24‐well culture plate with titanium sheets, and cultured for 12 h at 37 °C. The BHI (Brain Heart Infusion) medium was replaced with different material leach liquors and incubated for 12 h at 37 °C. The bacterial biofilms on the titanium surface were fixed by 2.5% glutaraldehyde for 30 min, and dehydrated using gradient concentrations of ethanol (30%, 50%, 70%, 80%, 90%, 95%, and 100%) for 15 min, respectively. Then, the samples were dried and observed under SEM.

### Hemolysis Test and Blood Cell Adhesion

The hemocompatibility of the ColTA powder adhesive was evaluated by a hemolysis test of rat blood. The rat blood was collected by heparin anticoagulant tubes, and centrifuged to obtain erythrocytes at 2000 rpm for 10 min. The collected erythrocytes were washed with normal saline three times and prepared to 2% (v/v) erythrocyte saline solution. Respectively, Col‐II, TA, and ColTA powder (50 mg) were mixed with the erythrocyte saline solutions and incubated at 37 °C for 24 h. Normal saline and 0.1% Triton X‐100 were separately set as negative control and positive control. The erythrocyte saline solutions were centrifuged at 5000 rpm for 15 min, and the optical densities (OD) of the supernatants at 570 nm were measured with a microplate reader (SPECTROstar Nano, BMG Labtech, Offenburg, Germany). The hemolysis ratios were calculated according to the following formula: Hemolysis ratio (%) = (OD_sample_ – OD_normal saline_)/ (OD_Triton_ – OD_normal saline_) × 100%.

ColTA adhesives were prepared in cylindrical molds. The anticoagulated whole blood was dripped onto the ColTA adhesive and incubated at 37 °C for 5 min. After washing with PBS three times, the samples were fixed with 2.5 wt.% of glutaraldehyde for 24 h, and then dehydrated by graded concentrations of ethanol (30%, 50%, 70%, 90%, 95%, and 100%) for 10 min, respectively. The samples were dried at 37 °C overnight and the blood cell adhesion was observed with SEM.

### In Vitro BCI Determination

Sixty microliter calcium chloride aqueous solution (0.1 m) was added to 540 µL heparinized rat blood. 20 mg ColTA powder was added into the wells of 96 well plates, and 50 µL CaCl_2_‐activated blood was dripped into the wells with or without ColTA powder. The unclotted blood was washed away softly using normal saline at different time points. The OD values of eluents at different time points were measured by an ultraviolet spectrophotometer at 540 nm. The OD value of 5 mL of normal saline containing 50 µL blood was regarded as a reference group. The BCI (%) was calculated according to the following equation: BCI = ODs/ODr × 100% (ODs: OD values of the samples, ODr: OD values of the reference).

### Evaluation of Hemostatic Effect In Vivo

Wistar rats (male, 8 weeks) were used for the hemostatic test approved by the Medical Ethics Committee of Stomatological Hospital, Shandong University, Jinan, China (Permit Number: No. 20220310) and based on the ethical guidelines of the Care and Use of Laboratory Animals of the Chinese Science and Technology Ministry. The rats were anesthetized by inhaling isoflurane (RWD, Shenzhen, China) with a small animal anesthesia machine during the surgical operation. The hepatic hemorrhage model of the rat was established to estimate the hemostatic performance of ColTA powder according to a previous study.^[^ ^6^ ^]^ Abdominal incisions were made in rats to expose the liver. The filter paper was weighed in advance and placed under the liver. Then, the liver was cut with a 0.5 cm long wound, and 100 mg ColTA powder was applied to the wound. The control group was not treated. The weight of the filter paper was recorded again to calculate blood loss after absorbing blood. In addition, the cardiac, tail vein, and femoral artery bleeding models of rats were also established to further evaluate the hemostatic effect of ColTA powder. Instantly, the ColTA powder was scattered over the bleeding spots as soon as bleeding after piercing the heart, femoral artery, and cutting the tail vein.

### RNA Isolation and qRT‐PCR

RAW 264.7 macrophages were cultured with α‐minimum essential medium (α‐MEM, BioInd, Kibbutz, Israel) containing 10% fetal bovine serum (FBS, Gibco, Carlsbad, USA). RAW 264.7 cells were inoculated to 6 well cell culture plates, and the next day the medium was replaced with material leach liquors containing LPS (1 µg mL^−1^; Solarbio) for 24 h. The total RNA of the RAW 264.7 cells was isolated using TRIzol reagent (Takara, Kusatsu, Japan) by the manufacturer's protocols. The concentration and purity of RNA for each sample were measured by Nanodrop 2000 ultramicro spectrophotometer (Thermo Fisher Scientific, Waltham, MA, USA). Then, RNA was reverse‐transcribed to complementary DNA with the PrimeScript RT reagent kit (Takara) according to the instructions. Quantitative real‐time PCR assays were conducted by using SYBR Premix Ex Taq II (Takara) with LightCycler 96 Real‐Time PCR System (Roche, Basel, Switzerland) to assess the gene expression of IL−1β, TNF‐α, NOS−2, CD86, Arg−1, and CD206. The primer sequences for amplifying target genes and housekeeping gene glyceraldehyde‐3‐phosphate dehydrogenase (GAPDH) were listed in Table S3 (Supporting Information).

### Immunocytochemistry

RAW 264.7 cells were inoculated to 6 well cell culture plates, and the next day the medium was replaced with material leach liquors containing LPS (1 µg mL^−1^) for 24 h. The cells were fixed with 4% paraformaldehyde for 15 min and then permeabilized by 0.5% Triton X‐100 for 15 min. After three washes with PBS, cells were blocked using 10% normal goat serum. Subsequently, cells were incubated with anti‐CD206 (host: rabbit) and anti‐iNOS (host: mouse) primary antibodies (1:500; Abcam, Cambridge, UK) at 4 °C overnight, washed with PBS and incubated with Alexa Fluor 594‐conjugated goat anti‐rabbit IgG secondary antibody and Alexa Fluor 488‐conjugated goat anti‐mouse IgG secondary antibody (1:500; Proteintech, Wuhan, China) in the dark for 1 h at 37 °C. Nuclei were redyed using 2‐(4‐Amidinophenyl)−6‐indolecarba midine dihydrochloride (DAPI; Solarbio). Images were observed under a fluorescence microscope (Leica DMi8, Wetzlar, Germany) in the darkroom.

### Protein Isolation and Western Blotting

RAW 264.7 cells were inoculated to 6 well cell culture plates, and the next day the medium was replaced with material leach liquors containing LPS (1 µg mL^−1^) for 1 h. The cells were lysed with RIPA lysis buffer (Solarbio) containing 1% phenylmethanesulfonyl fluoride (Solarbio) and 1% phosphatase inhibitor (Boster, Wuhan, China), and the whole protein was collected using cell scrapers. The concentration of protein was measured by a BCA Protein Assay Kit (Solarbio). 10% SDS‐PAGE gels were used to separate equal loading quantity of protein (20 µg/lane) and then the proteins of different molecular weights were transferred onto the polyvinylidene fluoride membranes (Millipore, Billerica, MA, USA). After blocking with 5% nonfat milk for 1 h, the membranes were probed with the primary antibodies overnight at 4 °C and incubated with horseradish peroxidase‐conjugated secondary antibodies (1:5000; Proteintech, Chicago, IN, USA) for 1 h at room temperature. The protein bands were visualized with enhanced chemiluminescence reagents (Millipore) and scanned using an extra‐sensitive imager (Amersham Imager 600; GE Healthcare Life Sciences, Pittsburgh, PA, USA). The protein expression level was quantified using Image J software. The primary antibodies and dilution ratios were as follows: rabbit anti‐NF‐κB p65 (1:1000; Abcam), rabbit anti‐IκBα (1:1000; Abcam), rabbit anti‐phospho‐NF‐κB p65 (1:1000; Abcam), rabbit anti‐phospho‐IκBα (1:1000; Abcam), and GAPDH (1:5000; Proteintech).

### Rat Infectious Skin Defect Model Preparation, Biosecurity, and Wound Healing Evaluation

Wistar rats (male, 8 weeks) were used for the wound healing test approved by the Medical Ethics Committee of Stomatological Hospital, Shandong University, Jinan, China (Permit Number: No. 20220310). The rats were anesthetized by inhaling isoflurane (RWD, Shenzhen, China) using a small animal anesthesia machine. Full‐thickness round skin defects (diameter: 8 mm) were prepared using punchers on the back of rats. The silicone gaskets with 22 mm outside diameter, inside diameter 12 mm, and thickness 1 mm were fixed around the circular skin defects to reduce skin contraction during the healing process. *S. aureus* was dripped onto each wound to acquire the infectious models with 10 µL bacterial suspension (1 × 10^8^ CFU mL^−1^). Then, the wounds were treated with ColTA powder, covered by transparent dressings (3 m Tegaderm, St. Paul, USA), and wrapped using elastoplasts (3 m). The defect without powder was considered as the control group. The wounds were photographed to record the healing process at days 0, 5, 9, and 14 after surgery. The healing rate (%) was calculated according to the following equation: Wound healing ratio = (A_O_ − A_C_)/(A_O_) × 100% (A_O_: the area of the original wound, A_C_: the area of the current wound).

The rats were euthanized by taking overdose anesthesia at days 7 and 14 after surgery. Peripheral blood of rats at days 14 after surgery was collected for blood cell analysis. The skin and internal organ samples were collected and fixed with 4% paraformaldehyde for 48 h. Afterward, the fixed samples underwent dehydration with gradient concentrations of ethanol, vitrification with dimethylbenzene, paraffin embedding, and tissue section (5 µm thickness). The tissue slices were treated with H&E staining, Masson staining, and immunohistochemical staining for α‐SMA (Abcam) to assess the wound healing and angiogenesis according to the manufacturer's introductions. Immunofluorescence double staining for CD206 (Abcam) and iNOS (Abcam) was used to evaluate the immune response in wounds. The dermal gaps were measured by Image J software, and the number of CD206+ or iNOS+ cells and microvessels were also counted with ImageJ software.

### Statistical Analysis

Data were presented as mean ± standard deviation (SD). Statistical variances between the two groups were analyzed by two‐way *t*‐test using GraphPad Prism software (version 6; MacKiev Software, Boston, MA, USA). *p* < 0.05 was identified as a statistically significant difference.

## Conflict of Interest

The authors declare no conflict of interest.

## Supporting information

Supporting Information

Supplemental Video 1

Supplemental Video 2

Supplemental Video 3

## Data Availability

The data that support the findings of this study are available from the corresponding author upon reasonable request.
