# Supplementary material for: Hydro‐Sensitive, In Situ Ultrafast Physical Self‐Gelatinizing, and Red Blood Cells Strengthened Hemostatic Adhesive Powder with Antibiosis and Immunoregulation for Wound Repair
Source: Adv Sci (Weinh). 2023 Nov 30;11(4):2306528. doi: 10.1002/advs.202306528 (PMC10811473; doi:10.1002/advs.202306528)
Supplement: Supplementary file 1 — Supporting Information [file ADVS-11-2306528-s003.pdf]

ADVANCED SCIENCE
Open Access

Supporting Information

for *Adv. Sci.*, DOI 10.1002/advs.202306528

Hydro-Sensitive, In Situ Ultrafast Physical Self-Gelatinizing, and Red Blood Cells Strengthened Hemostatic Adhesive Powder with Antibiosis and Immunoregulation for Wound Repair

Lingling Shang, Yonggan Yan, Zhao Li, Hong Liu, Shaohua Ge* and Baojin Ma**

Hydro-sensitive, in situ Ultrafast Physical Self-gelatinizing, and Red Blood Cells Strengthened Hemostatic Adhesive Powder with Antibiosis and Immunoregulation for Wound Repair

Lingling Shang[1#], Yonggan Yan[1#], Zhao Li[1], Hong Liu[2*], Shaohua Ge[1*], Baojin Ma[1*]

[1] Department of Periodontology & Tissue Engineering and Regeneration, School and Hospital of Stomatology, Cheeloo College of Medicine, Shandong University & Shandong Key Laboratory of Oral Tissue Regeneration & Shandong Engineering Laboratory for Dental Materials and Oral Tissue Regeneration & Shandong Provincial Clinical Research Center for Oral Diseases, Jinan, Shandong, 250012, China

2 State Key Laboratory of Crystal Materials, Shandong University, Jinan, Shandong, 250013, China

[#] These authors contributed equally.

Corresponding Authors: baojinma@sdu.edu.cn (Baojin Ma); shaohuage@sdu.edu.cn (Shaohua Ge); hongliu@sdu.edu.cn (Hong Liu).

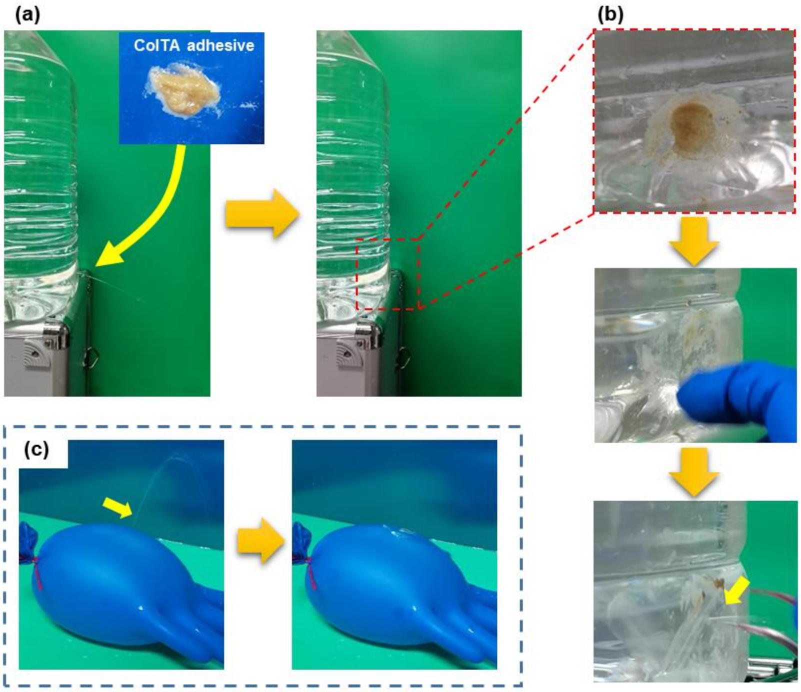

Figure S1 Pressure tests of the ColTA adhesive. **(a)** Pressure test of the adhesive using a damaged plastic bottle filled with water. **(b)** The stability of the adhesive-repaired area. **(c)** Pressure test of the adhesive using a water-filled balloon.

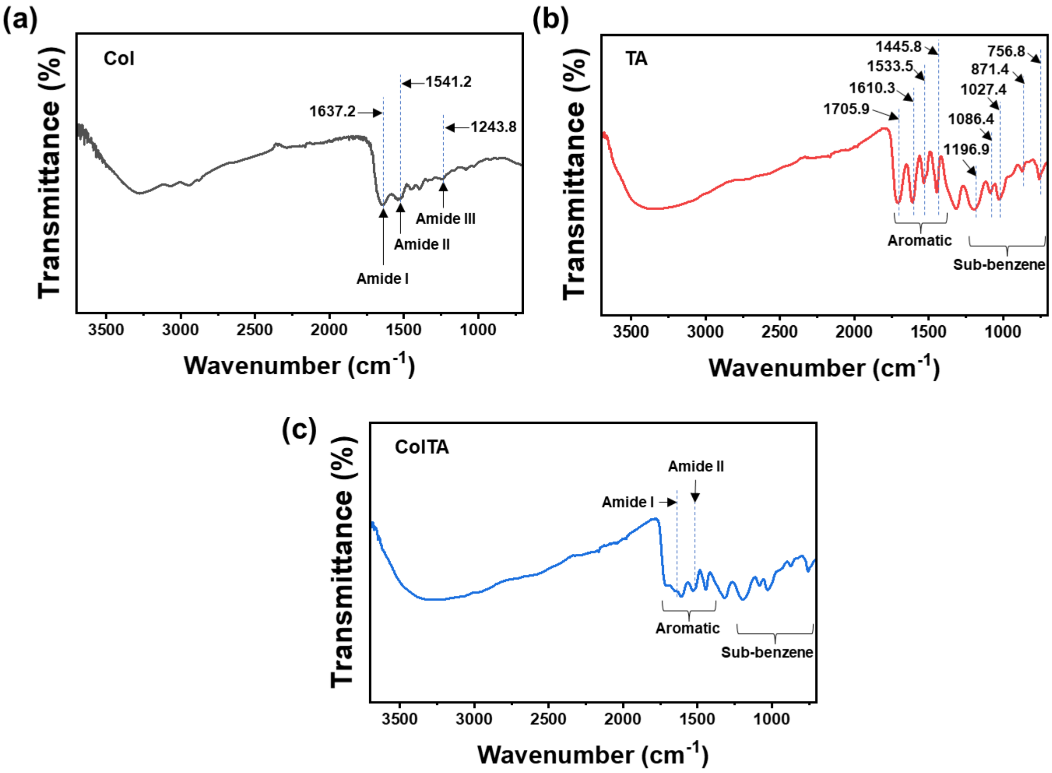

Figure S2 FTIR spectra of **(a)** Col, **(b)** TA and **(c)** ColTA(water) adhesive.

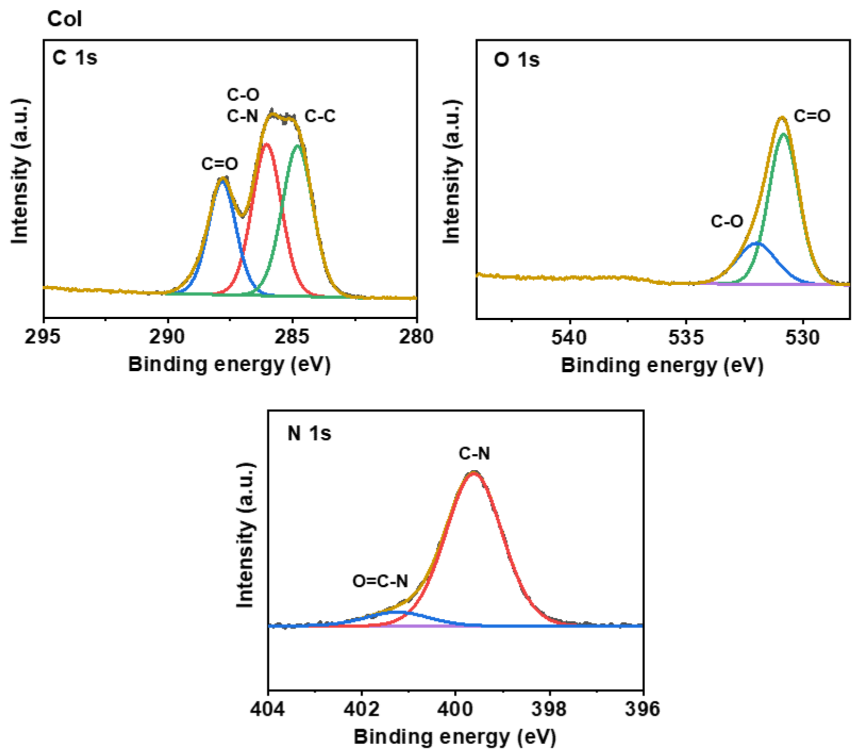

Figure S3 High-resolution spectra of C 1s, O 1s and N 1s for the Col. All XPS spectra are plotted after shifting C1s to 284.8 eV.

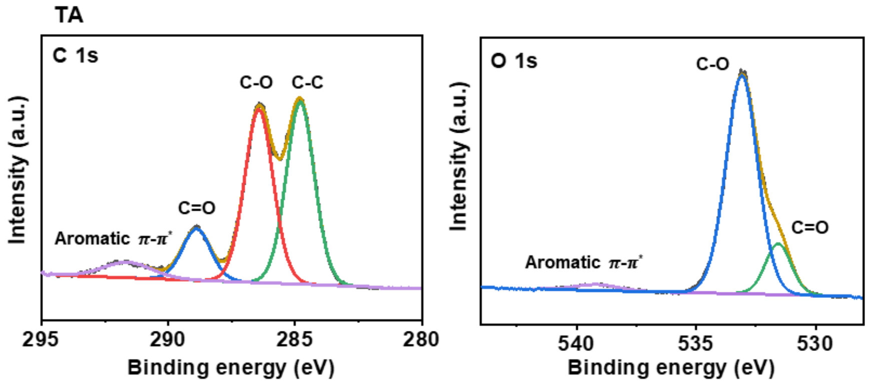

Figure S4 High-resolution spectra of C 1s and O 1s for the TA. All XPS spectra are plotted after shifting C1s to 284.8 eV.

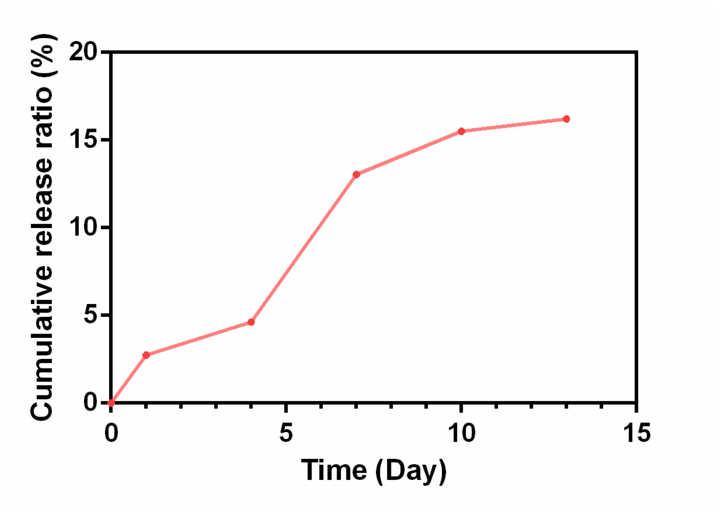

Figure S5 The cumulative release rate curve of TA from ColTA in leachate.

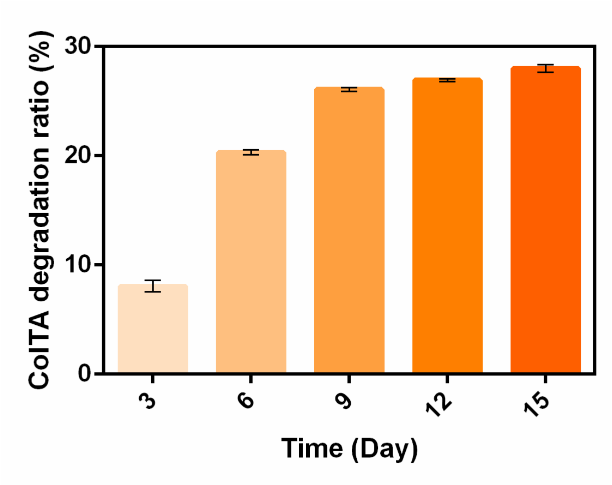

Figure S6 The degradation ratio of ColTA adhesive in PBS.

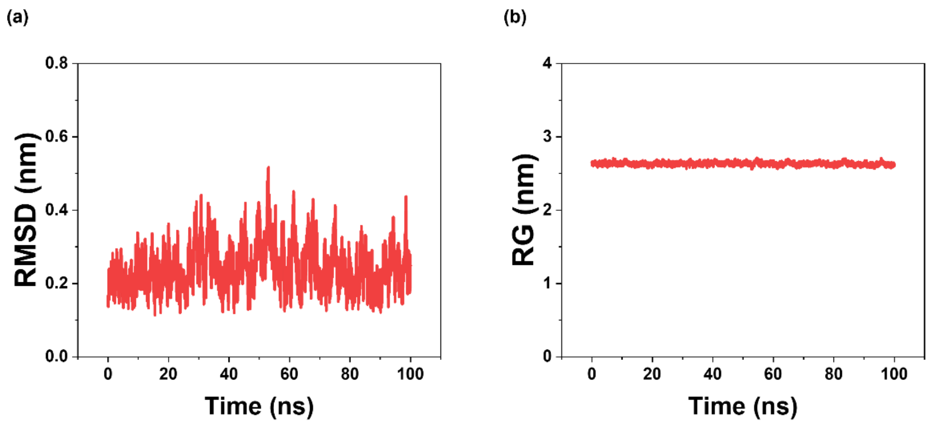

Figure S7 (a) Root mean square deviation (RMSD) of Col-II group and ColTA complex group during MDS. **(b)** Radius of gyration (Rg).

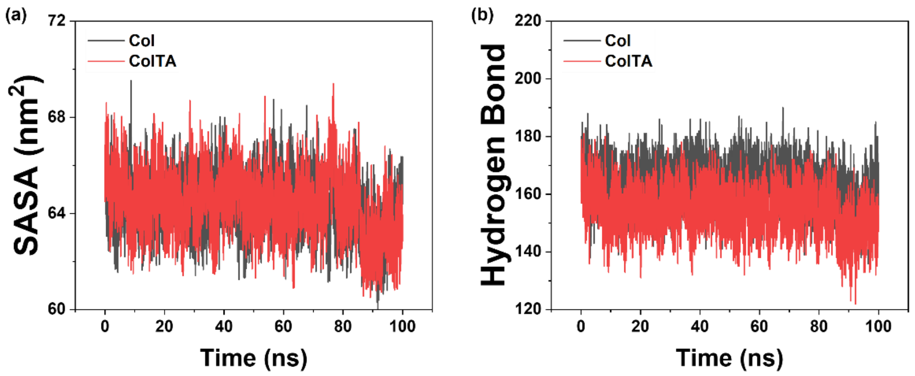

Figure S8 (a) Protein solvent-accessibility surface area (SASA). **(b)** The number of hydrogen bonds between Col-II and water in aqueous solution.

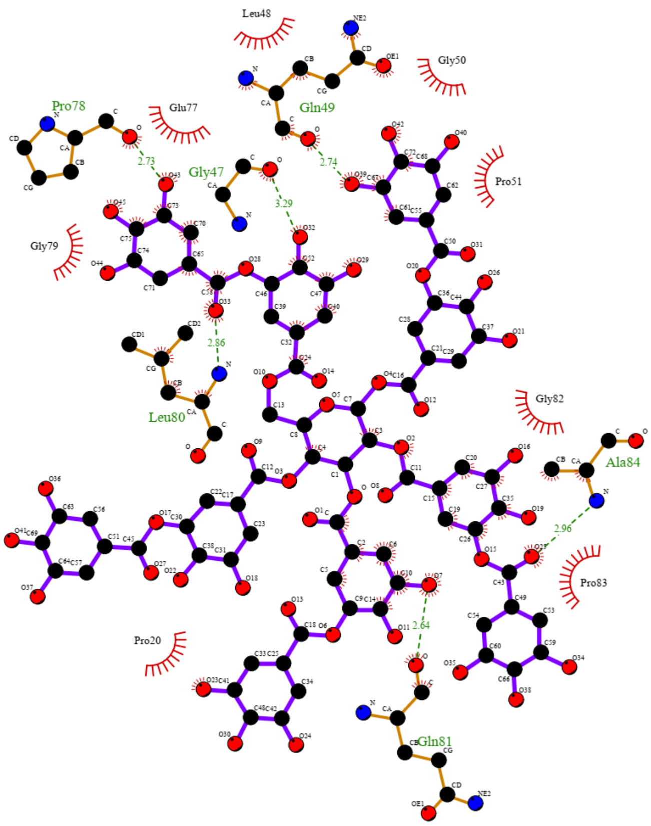

Figure S9 The detailed results of H-bonds between Col-II and TA after molecular docking.

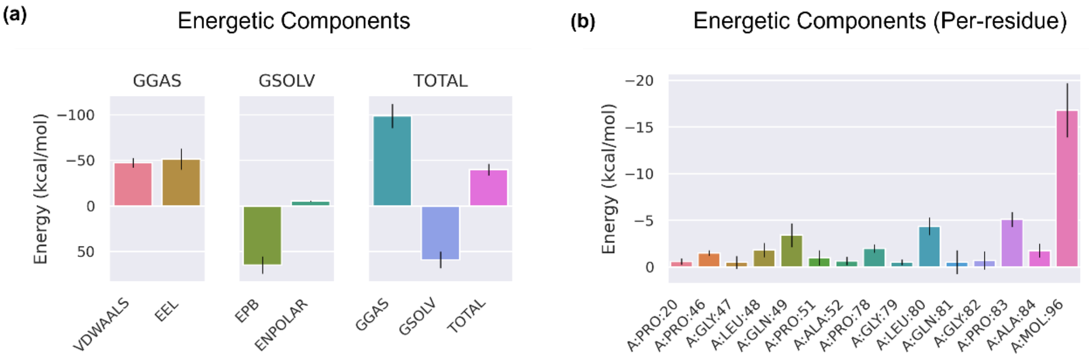

Figure S10 Binding free energy. (a) Attribute decomposition, **(b)** Binding sites decomposition. VDWAALS: van der Waals energy; Eel: Electrostatic energy; EPB: Polar solvation energy; ESURF: Non-polar solvation energy; GGAS: Total gas phase free energy; GSOLV: Total solvation free energy; TOTAL: GSOLV + GGAS.

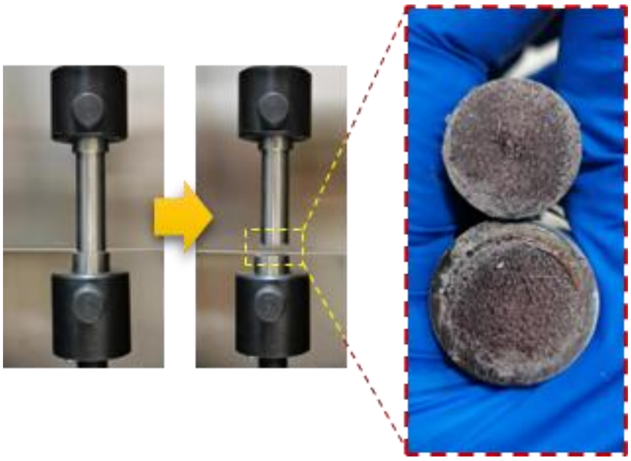

Figure S11 Photographs of the tensile adhesion tests and adhesive failure.

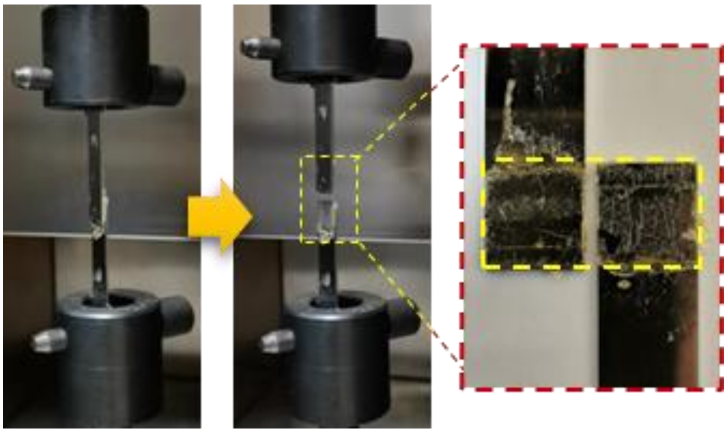

Figure S12 Photographs of the lap-shear adhesion tests and adhesive failure.

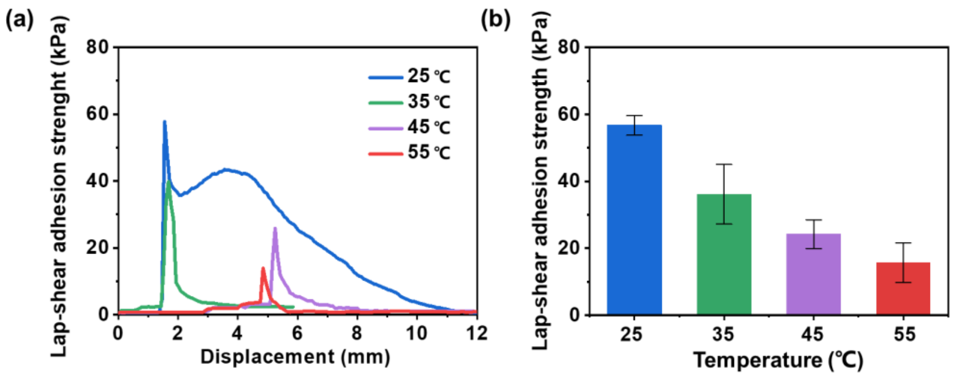

Figure S13 (a) Lap-shear adhesion curves and **(b)** strength of the adhesives on the iron substrate at different temperatures.

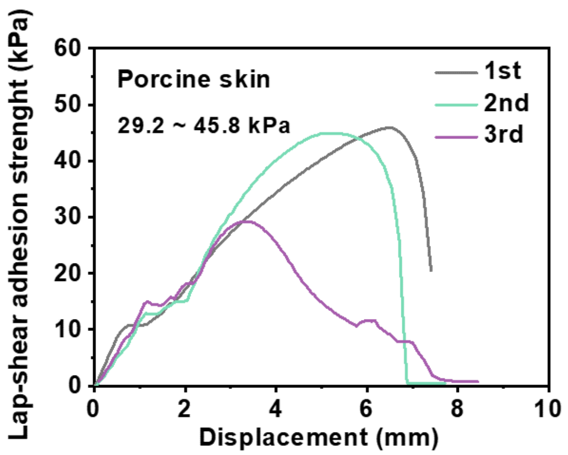

Figure S14 Lap-shear adhesion curves of the ColTA adhesive on porcine skin.

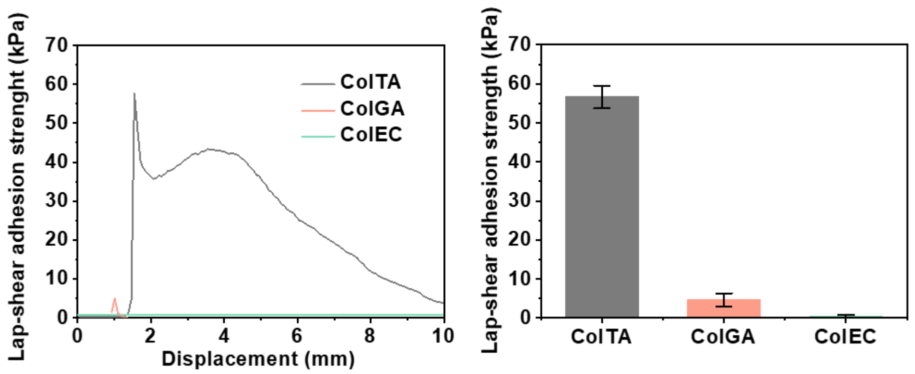

Figure S15 Lap-shear adhesion curves and strengths of Col-II incorporated with TA, GA, and EC (referred to as ColTA, ColGA, and ColEC).

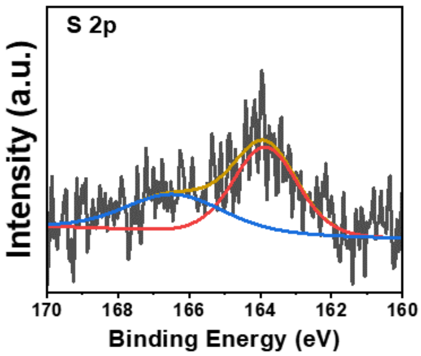

Figure S16 High-resolution spectra of S 2p for the ColTA (blood) adhesive. The XPS spectra is plotted after shifting C1s to 284.8 eV.

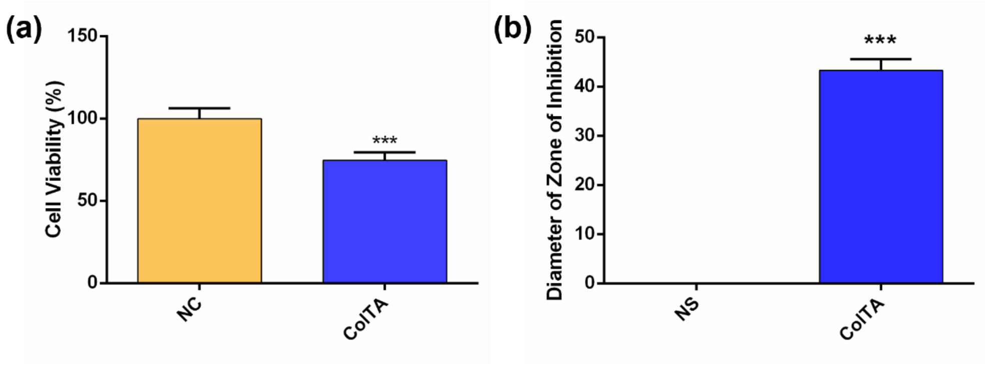

Figure S17 (a) Cell viability after cultivation with or without leach liquor from ColTA. **(b)** Bacteriostasis ring diameters of the ColTA adhesive.

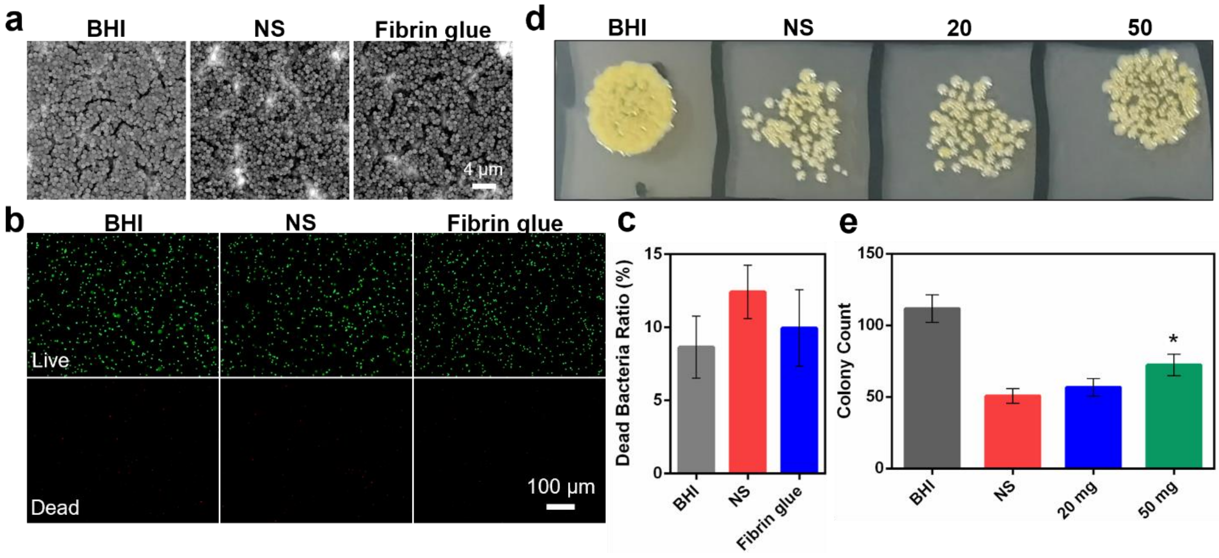

Figure S18 Antibacterial activity of fibrin glue. **(a)** Bacterial biofilms formed by *S. aureus* incubated with BHI, normal saline, and fibrin glue liquor on a titanium surface. **(b)** Live and dead bacteria staining of the *S. aureus*. **(c)** Counting for the ratios of dead bacteria. **(d)**

Photographs of bacterial colonies from *S. aureus* incubated with BHI, normal saline, and fibrin glue liquors. **(e)** Counting for the number of *S. aureus* colonies. * $P < 0.05$ compared with the NS group.

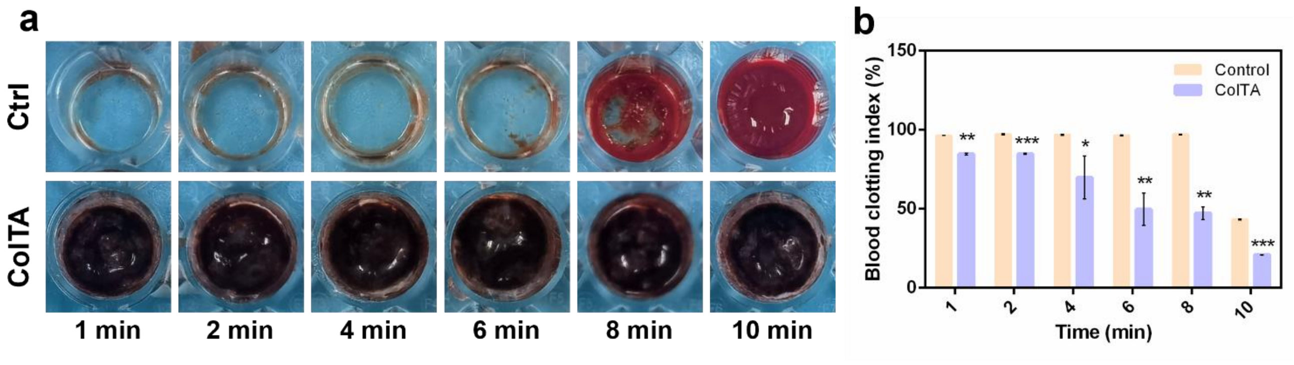

Figure S19 In vitro blood-clotting performance of ColTA powders. **(a)** Photos of blood clotting after adding ColTA powder to whole blood. **(b)** The corresponding blood clotting indexes.

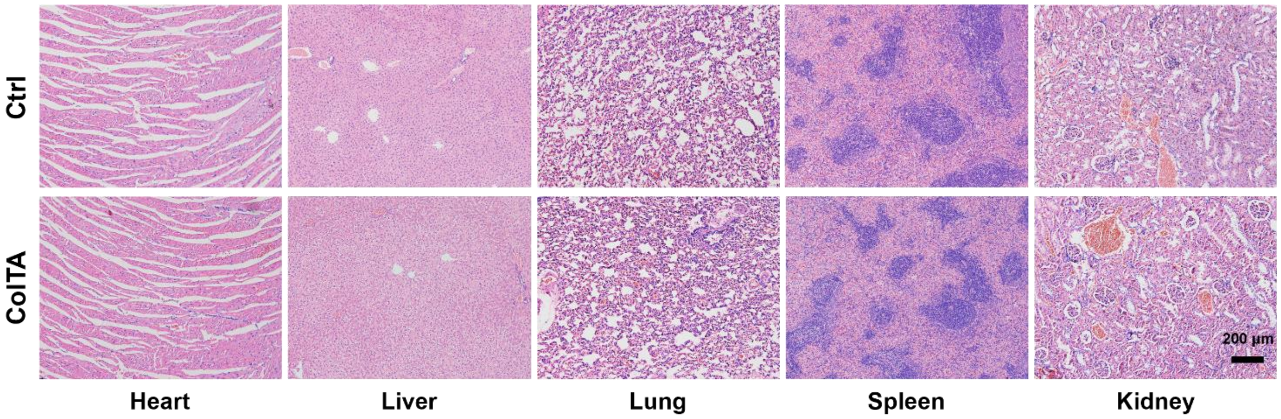

Figure S20 H&E staining of organs of the body. ColTA powder had no significant toxic effect on the organs of the body.

Table S1 The blood cell analysis of rats in the control group

Parameters	Abbreviation	Result	Unit	Reference range
White Blood Cell Count	WBC	8.1	10^9/L	1.9-16.8
Lymphocyte Count	Lymph#	6.0	10^9/L	0.91-12.2
Monocyte Count	Mon#	0.3	10^9/L	0.08-2.3
Neutrophil Count	Gran#	1.8	10^9/L	0.35-6.3
Lymphocyte Percentage	Lymph%	73.9	%	40-88.9
Monocyte Percentage	Mon%	3.7	%	2-18
Neutrophil Percentage	Gran%	22.4	%	7.3-50
Erythrocyte count	RBC	7.07	10^12/L	5-9.8
Hemoglobin	HGB	138	g/L	120-170
Hematocrit	HCT	41.3	%	32-53
Mean Corpuscular Volume	MCV	58.5	fL	50-67
Mean Corpuscular Hemoglobin	MCH	19.5	pg	16-23
Mean Corpuscular Hemoglobin Concentration	MCHC	334	g/L	300-370
Red Cell Distribution Width	RDW	11.6	%	11-16
Platelet Count	PLT	1084	10^9/L	250-1500
Mean Platelet Volume	MPV	7.2	fL	4.8-7.5
Platelet Distribution Width	PDW	16.8		12-17.5
Plateletocrit	PCT	.***	%	0.2-0.78

Table S2 The blood cell analysis of rats in the ColTA group

Parameters	Abbreviation	Result	Unit	Reference range
White Blood Cell Count	WBC	5.4	10^9/L	1.9-16.8
Lymphocyte Count	Lymph#	4.0	10^9/L	0.91-12.2
Monocyte Count	Mon#	0.2	10^9/L	0.08-2.3
Neutrophil Count	Gran#	1.2	10^9/L	0.35-6.3
Lymphocyte Percentage	Lymph%	74.6	%	40-88.9
Monocyte Percentage	Mon%	3.4	%	2-18
Neutrophil Percentage	Gran%	22.0	%	7.3-50
Erythrocyte count	RBC	6.96	10^12/L	5-9.8
Hemoglobin	HGB	137	g/L	120-170
Hematocrit	HCT	40.7	%	32-53
Mean Corpuscular Volume	MCV	58.6	fL	50-67
Mean Corpuscular Hemoglobin	MCH	19.6	pg	16-23
Mean Corpuscular Hemoglobin Concentration	MCHC	336	g/L	300-370
Red Cell Distribution Width	RDW	10.5	%	11-16
Platelet Count	PLT	1224	10^9/L	250-1500
Mean Platelet Volume	MPV	6.6	fL	4.8-7.5
Platelet Distribution Width	PDW	16.6		12-17.5
Plateletocrit	PCT	.***	%	0.2-0.78

Table S3 Primer sequences for qRT-PCR

Gene	Forward (5′-3′)	Reverse (5′-3′)
**IL-1β**	5′-CACCTTCTTTTCCTTCATCTT-3′	5′-TCACACACCAGCAGGTTATCATC-3′
**TNF-α**	5′-GGATCTCAAAGACAACCAAC-3′	5′-ACAGAGCAATGACTCCAAAG-3′
**Nos2**	5′-CAGAGGACCCAGAGACAAGC-3′	5′-TGCTGAAACATTTCCTGTGC-3′
**CD86**	5′-GGCCCTCCTCCTTGTGATG-3′	5′-CTGGGCCTGCTAGGCTGAT-3′
**Arg-1**	5′-CAGAAGAATG GAAGAGTCAG-3′	5′-CAGATATGCAGGGAGTV-3’
**CD206**	5′-CAGGTGTGGGCTCAGGTAGT-3′	5′-TGTGGTGAGCTGAAAGGTGA-3′
**GAPDH**	5’-TGACCACAGTCCATGCCATC-3’	5’-GACGGACACATTGGGGGTAG-3’
